# STAT6/LINC01637 axis regulates tumor growth via autophagy and pharmacological targeting STAT6 as a novel strategy for uveal melanoma

**DOI:** 10.1038/s41419-024-07115-5

**Published:** 2024-10-01

**Authors:** Bo Liu, Xueting Yao, Qinying Huang, Yichao Fan, Bo Yu, Jing Wang, Wencan Wu, Jinhui Dai

**Affiliations:** 1 Department of Ophthalmology, Zhongshan Hospital Affiliated to Fudan University, Shanghai, China; 2 The Eye Hospital, School of Ophthalmology &Optometry, Wenzhou Medical University, Wenzhou, China; 3 Department of Laboratory Medicine, Longhua Hospital, Shanghai University of Traditional Chinese Medicine, Shanghai, China; 4 Department of Ophthalmology, Peking University Shenzhen Hospital, Shenzhen, China

**Keywords:** Eye cancer, Autophagy

## Abstract

Compelling evidence has revealed a novel function of the STAT pathway in the pathophysiology of uveal melanoma (UM); however, its regulatory mechanisms remain unclear. Here, we analyzed the clinical prognostic value of STAT family genes in UM patients using bioinformatics approaches and found that high STAT6 expression is associated with poor prognosis. Furthermore, cellular experiments and a nude mouse model demonstrated that STAT6 promotes UM progression through the autophagy pathway both in vivo and in vitro. Next, RIP-PCR revealed that STAT6 protein binds to LINC01637 mRNA, which in turn regulates STAT6 expression to promote UM growth. Finally, molecular docking indicated that STAT6 is a target of Zoledronic Acid, which can delay UM tumorigenicity by inhibiting STAT6 expression. Taken together, our results indicate that the STAT6/LINC01637 axis promotes UM progression via autophagy and may serve as a potential therapeutic target for UM.

## Introduction

Uveal melanoma (UM) is a rare and aggressive form of melanoma that originates in the uveal tract of the eye. With an annual incidence of ~5 to 6 cases per million individuals, UM comprises roughly 5% of all melanoma cases [1]. Although UM is a rare condition, it is the most prevalent kind of primary intraocular malignancy in adults. It poses a substantial risk of metastasis, especially to the liver, which generally results in unfavorable outcomes [2, 3]. While these treatments have successfully controlled the primary tumor, they do not tackle the systemic nature of the disease, and the five-year survival rate for patients with metastatic UM remains low [4]. The limitations in current UM research are multifaceted. Firstly, the rarity of the disease poses a significant challenge for conducting large-scale clinical trials, which are essential for developing effective systemic therapies [5]. Secondly, the genetic heterogeneity of UM adds complexity to the disease, as different patients may present distinct molecular profiles that could influence treatment response [6]. Moreover, recent advances in genomic and molecular profiling have begun to elucidate the genetic landscape of UM. High-throughput sequencing has identified mutations in genes such as GNAQ and GNA11, prevalent in the majority of UMs, and believed to play a crucial role in tumor initiation and progression [7]. However, the functional consequences of these mutations and their therapeutic implications are still under investigation. In summary, there is an urgent need to further explore new biomarkers for predicting the prognosis of UM patients, develop novel therapies targeting the causative genes, and establish a unique personalized therapeutic strategy for each UM patient.

The signal transducers and activators of transcription (STATs) play a pivotal role in cellular signal transduction pathways, especially in response to cytokines and growth factors. The STAT pathway is integral to various cellular processes, including cell growth, differentiation, apoptosis, and immune responses. Over the past few decades, research into the STAT family has expanded our understanding of its role in various diseases, especially in the context of cancer [8]. The STAT family includes seven members, namely STAT1, STAT2, STAT3, STAT4, STAT5A, STAT5B, and STAT6, each exhibiting unique and partially shared functionalities. They are activated through a phosphorylation event that triggers a conformational change, allowing the STAT proteins to homo- or heterodimerize, translocate to the nucleus, and regulate gene expression. This activation is tightly regulated to maintain cellular homeostasis; however, dysregulation of the STAT pathway has been implicated in numerous diseases [9]. For example, persistent activation of STAT3 has been observed in various cancers, including breast [10], ovarian [11], and head and neck cancers [12]. STAT3’s role in tumorigenesis is multifaceted: it can promote cell cycle progression, inhibit apoptosis, and stimulate angiogenesis. Additionally, it can modulate the tumor microenvironment to facilitate immune evasion, making it a promising target for therapeutic intervention [13]. STAT1, traditionally viewed as a mediator of interferon responses and a tumor suppressor, has also been shown to exhibit oncogenic properties under specific conditions [14]. STAT4 has been linked to an increased risk of developing autoimmune diseases such systemic lupus erythematosus and rheumatoid arthritis. In these conditions, the immune system becomes excessively activated, causing long-term inflammation and tissue damage [15]. The study of STAT6 has provided insights into its role in mediating the effects of interleukins IL-4 and IL-13, which are critical for Th2 cell differentiation and immune responses. Dysregulation of STAT6 has been linked to conditions such as allergies and asthma, where an overactive Th2 response contributes to disease pathology [9]. In conclusion, the STAT family of proteins represents a critical node in cellular signaling pathways with wide-ranging implications for human health and disease. Continued research into the roles of STAT proteins in various diseases is crucial for developing clinical therapies that can improve patient outcomes.

Studies have demonstrated that some members of the STAT family, such as STAT3, control the activation of genes related to cell growth, formation of new blood vessels, and the spread of cancer cells. Moreover, the interaction between STAT3 and other cancer-causing pathways introduces intricacy to the formation of tumors, since the exchange of signals may possibly enhance the cancer-causing effects [13]. Importantly, our previous data indicated that SOCS3, as a suppressor of the STAT signaling pathway, could inhibit UM growth through the autophagy pathway [16]. Consequently, we constructed a risk model of STAT family genes to assess the prognosis of UM patients. Intriguingly, STAT family genes can serve as prognostic biomarkers for UM, especially STAT6. Therefore, we would like to further explore the function of STAT6 in the progression of UM. STAT6 plays a crucial role in coordinating cellular responses, particularly those influenced by cytokines such as IL-4 and IL-13. STAT6, functioning as a transcription factor, has a crucial role in controlling gene expression. Its abnormal regulation has been linked to several physiological and pathological disorders [9]. STAT6 is a vital component of the immune system that is responsible for guiding the transformation of naïve CD4 + T cells into Th2 cells. These Th2 cells are critical for mounting an immune response against extracellular parasites and for triggering allergic responses [17]. In addition to its involvement in immunological responses, STAT6 is also involved in the control of several other biological processes. For instance, STAT6 plays a role in the development and function of the nervous system, being involved in processes like neurogenesis and synaptic plasticity [18]. In oncology, STAT6 has emerged as a potential factor in tumor biology, with growing evidence suggesting its involvement in tumorigenesis and metastasis. For instance, STAT6 is implicated in regulating epithelial-mesenchymal transition (EMT) [19]. Moreover, STAT6 can influence the tumor microenvironment by modulating immune cell activity, potentially promoting immune evasion and tumor progression [20]. Nevertheless, our comprehension of STAT6’s role in health and disease is incomplete. The complexity of STAT6’s functions and its interactions with other signaling pathways pose significant challenges for researchers. Unraveling the precise molecular mechanisms through which STAT6 contributes to disease pathogenesis is crucial for developing targeted therapies.

In this study, we identified that STAT family genes possess prognostic value in UM patients. Furthermore, STAT6 promotes UM progression by inhibiting autophagy. Additionally, STAT6 binds to LINC01637 mRNA, which in turn promotes UM growth by regulating STAT6 expression. Finally, Zoledronic Acid targeted and inhibited STAT6 expression, thereby reducing UM tumorigenicity. Our study elucidated the molecular mechanism of STAT6 in UM, providing a new theoretical basis for targeted UM therapies.

## Material and methods

### Analysis of online database

The STAR-counts data and clinical information for UM were obtained from the TCGA dataset repository (https://portal.gdc.com). TPM format was utilized to extract the data, and log2(TPM + 1) was employed to normalize the data. Samples with RNA-seq data and clinical information were retained for subsequent analysis, and the bioinformatic analysis methods were previously described [21, 22].

### Cell lines and culture

The UM cell lines 92.1 and MUM-2B were obtained from the FuHeng Cell Center in Shanghai, China. The cells were cultured in RPMI 1640 media (Thermo Fisher, C11875500BT) supplemented with 10% fetal bovine serum (FBS) (Lonicera, S711-001S). Genetic profiling using STR analysis was conducted on all cell lines. The main uveal melanocyte tissues were obtained from our prior work [16, 23].

### Western blotting and quantitative real-time PCR

The procedures for Western blotting and quantitative real-time PCR were as previously reported [24]. For Western blotting, the primary antibodies used were anti-GAPDH (1:1000; Bioss, bsm-0978M), anti-STAT6 (1:1000; Beyotime, AF1534), anti-LC3 (1:1000; Abcam, ab192890), anti-P62 (1:1000; Abcam, ab109012). The primers for qPCR were as follows: GAPDH: F: AATGGGCAGCCGTTAGGAAA, R: GCCCAATACGACCAAATCAGAG; STAT6: F: CTCGCTGGACAGAGCTACAG, R: GACTTGGAGGTTGCCTCGGA; STAT5B: F: ACATTAAGGCCACCCAGCTC, R: AGCGGTCATACGTGTTCTGG; STAT5A: F: GTCACGCAGGACACAGAGAA, R: TGGGCAAACTGAGCTTGGAT; STAT2: F: AGACCAGAACTGGCAGGAAG, R: GATCCTGAATGTCCCGGCAG; STAT1: F: CAGCTGCAGAACTGGTTCAC, R: CATGCAGGGCTGTCTTTCCA; LINC01637: F: TTACTCATGTCCCCGCATCG, R: TCTCTGCGTTGTAGGTAGACC. The RNA Immunoprecipitation Kit (#Bes5101, BersinBio, China) was used in accordance with the manufacturer’s instructions. RIP-qPCR was performed as described previously [25].

### Cell proliferation assays and cell treatment

A total of 2000 cells per well were seeded in 96-well plates, with the cells being 92.1 and MUM-2B. Each well was exposed to the Cell Counting Kit-8 (CCK-8) reagent (1:11; Beyotime, C0038) for a period of two hours. The optical density at 450 nm was then measured. UM cells were cultured in RPMI 1640 medium with 10% FBS and treated with 30 μmol/L rapamycin (Apexbio, A8167). Zoledronic Acid was purchased from the MCE company (MCE, 118072-93-8). The plasmids for STAT6 and LINC01637 were synthesized by GenePharma (Shanghai, China).

### Transwell migration and invasion assay

To evaluate the migratory and invasive capabilities of UM cells, we employed the 24-well Transwell system (Corning, 3422, 354480) in accordance with established procedures. The cells, 1 × 10^4^ 92.1 and MUM-2B, were suspended in serum-free RPMI 1640 medium and plated in the upper chamber. The lower chambers were subsequently filled with RPMI 1640 medium supplemented with 20% FBS, a key component in cell culture. Following an interval of 24 h, the cells were fixed for 10 min in 4% paraformaldehyde (PFA), after which they were stained for a further 10 min with crystal violet (Beyotime, C0121).

### Xenografts assays

Four-to six-week-old BALB/c nude mice were obtained from Charles River Laboratories in China and were maintained at Longhua Hospital’s SPF laboratory animal facility. Mice were injected subcutaneously with 2 × 10^6^ UM cells grown in 100 μL PBS. Tumor size was assessed weekly using a caliper, and tumor volume was computed using techniques from the literature [16]. After four weeks, the mice were euthanized, and the tumors were excised and weighed. Photographs were also taken.

### Statistical analysis

The results of the experiment were presented as the mean ± standard deviation. The statistical analysis was conducted using GraphPad Prism 8, with Student’s *t* test employed for two-group comparisons and one-way or two-way ANOVA with Bonferroni post hoc tests for multiple groups. The threshold for statistical significance was set at *p* < 0.05.

## Results

### Prognostic value of STAT family genes in UM

**Fig. 1:**
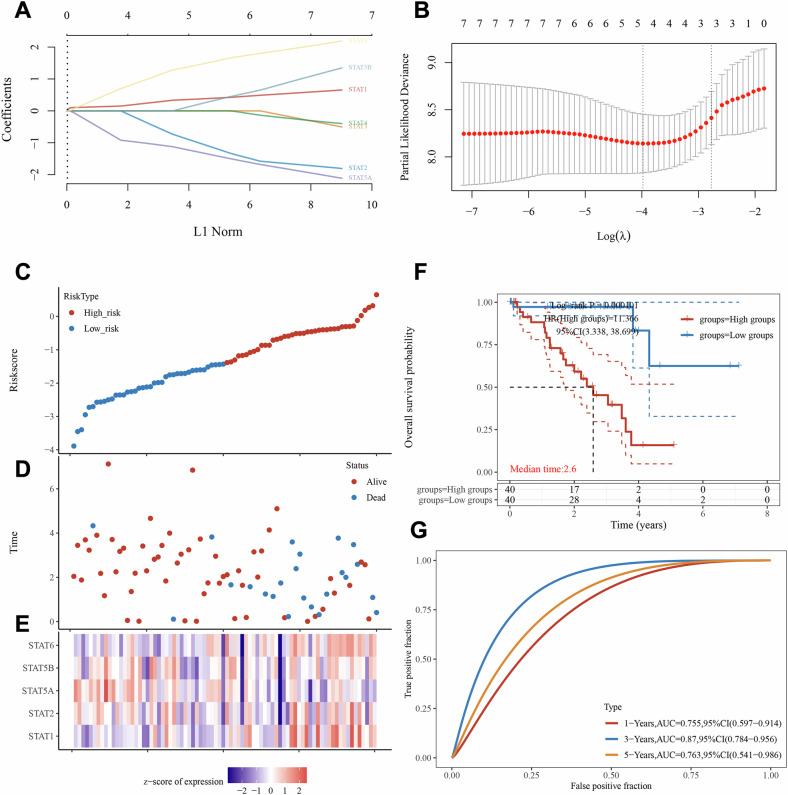
Prognostic value of STAT family genes in UM. **A** LASSO coefcients profles of 7 STAT family genes; (**B**) Using the minimum lambda value, LASSO regression with tenfold cross-validation identified 5 prognostic genes; (**C**) Distribution of STAT family gene model-based risk score; (**D**) Differences between high-risk and low-risk groups in survival status and survival time; (**E**) Heatmap of 5 prognostic gene expression profiles in low and high risk groups; (**F**) Kaplan–Meier survival curves for OS of patients in high and low risk group; (**G**) Time-dependent ROC analysis the of STAT family gene model.

Previous studies have shown that SOCS3 suppressed the growth of UM, and recognizing that SOCS3 is an important inhibitor of the STAT pathway, we aimed to further explore the function of the STAT pathway in UM [16]. Initially, we identified seven STAT family genes, followed by using LASSO Cox regression analysis to build a prognostic model using the expression profiles of these STAT family genes in UM patients, and identified five STAT family genes for the construction of the prognostic model based on the optimal value of the parameter λ (Fig. 1A, B). Figure 1C–E shows the distribution of the five STAT family genes in UM patients, the survival status, and the expression heat map across different groups. Subsequently, the ability of the model to predict the clinical prognosis of UM patients was determined by K-M survival curves and by analyzing the different survival times in the high and low-risk groups, and the results in Fig. 1F showed that the survival rate of UM patients in the high-risk group was significantly lower than that in the low-risk group. Finally, we used ROC curves at different times to assess the predictive performance of risk scores for OS, and the area under the curve reached 0.755 at 1 year, 0.870 at 3 years, and 0.763 at 5 years (Fig. 1G). The above data indicated the prognostic value of STAT family genes in UM.

### STAT6 promotes the progression of UM in vivo and in vitro

**Fig. 2:**
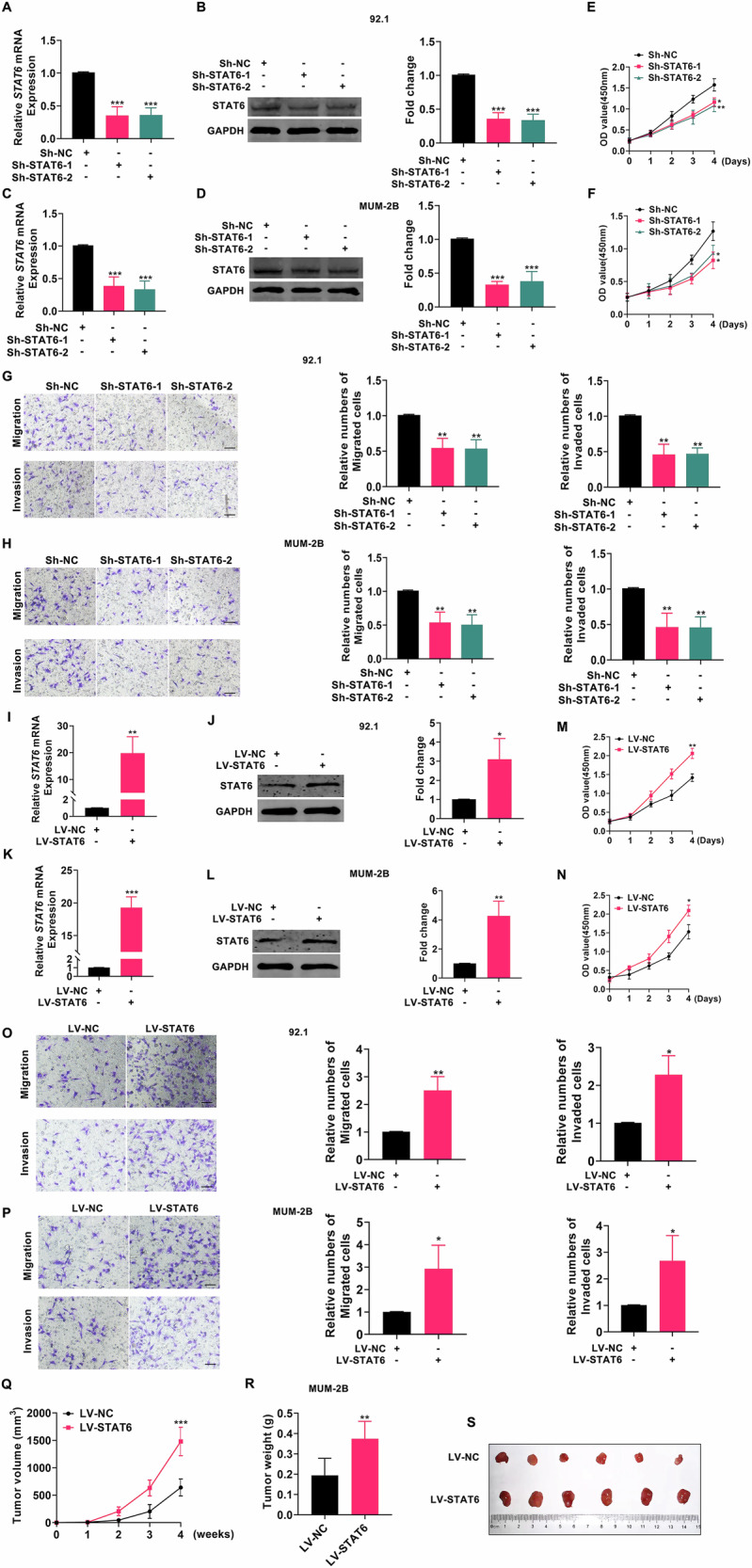
STAT6 promotes the progression of UM in vivo and in vitro. **A**, **C** The expression level of STAT6 mRNA in UM cells following STAT6 knockdown; (**B**, **D**) STAT6 knockdown was used to analyze the expression of STAT6 in UM cells through Western blotting; (**E**–**H**) This analysis examined how the knockdown of STAT6 affected the proliferation, migration, and invasion of UM cells.; (**I**, **K**) The level of STAT6 mRNA expression in UM cells that were overexpressing STAT6; (**J**, **L**) STAT6 overexpression in UM cells was analyzed by Western blotting.; (**M**–**P**) A comprehensive analysis of the proliferation, migration, and invasion of UM cells in vitro overexpressing STAT6; (**Q**) The volume of tumors formed in the LV-NC and LV-STAT6 groups was quantified; (**R**) The weight of tumors formed in the LV-NC group and the LV-STAT6 group were quantified. **S** The photographic images of tumors from the LV-NC and LV-STAT6 groups. (Scale bar: 100 µm; Data were presented as the mean ± SD; *n* = 3–6; *p < 0.05, **p < 0.01, ***p < 0.001).

RNA was extracted from uveal melanocytes (U-94) and UM cells (92.1 and MUM-2B), and the expression levels of the aforementioned five genes were determined via qPCR experiments. As depicted in Fig. S1A, STAT6 expression was significantly elevated in UM cells compared to the control group, while the expression differences of the other four genes in UM cells were inconspicuous. Therefore, STAT6 was selected for further investigation. Subsequent analysis of clinical data from UM patients revealed a poorer prognosis among those with high STAT6 expression (Fig. S1B), corroborated by the increased protein levels of STAT6 observed in UM cells (Fig. S1C). These findings suggest that STAT6 may play a pivotal role in UM progression. To further confirm STAT6’s role in UM, we conducted STAT6 gene knockdown experiments in UM cell lines (92.1 and MUM-2B), successfully achieving STAT6 knockdown in UM cells, as illustrated in Fig. 2A–D. Subsequently, we employed the STAT6 knockdown cells to conduct proliferation, migration, and invasion assays. Subsequently, we assessed the proliferation, migration, and invasion capabilities of UM cells following STAT6 overexpression. As depicted in Fig. 2I–L, UM cells exhibited successful STAT6 overexpression. Following STAT6 overexpression, UM cells displayed enhanced proliferation, migration, and invasion abilities compared to the control group, as illustrated in Fig. 2M–P. Subsequently, to further elucidate the impact of STAT6 on UM in vivo, UM cells stably overexpressing STAT6 and control cells were separately injected into the subcutaneous tissue of nude mice, and tumor formation volume was monitored weekly. As depicted in Fig. 2Q, the volume of tumor formation was significantly greater in the group overexpressing STAT6 compared to the control group. Subsequently, subcutaneous tumor specimens were collected from the mice after 4 weeks, and as illustrated in Fig. 2R, S and Fig. S2, the tumor weight was significantly greater in the group overexpressing STAT6 compared to the control group. The above data indicated that STAT6 promoted the growth of UM both in vivo and in vitro.

### STAT6 promotes the growth of UM cells by inhibiting autophagy

**Fig. 3:**
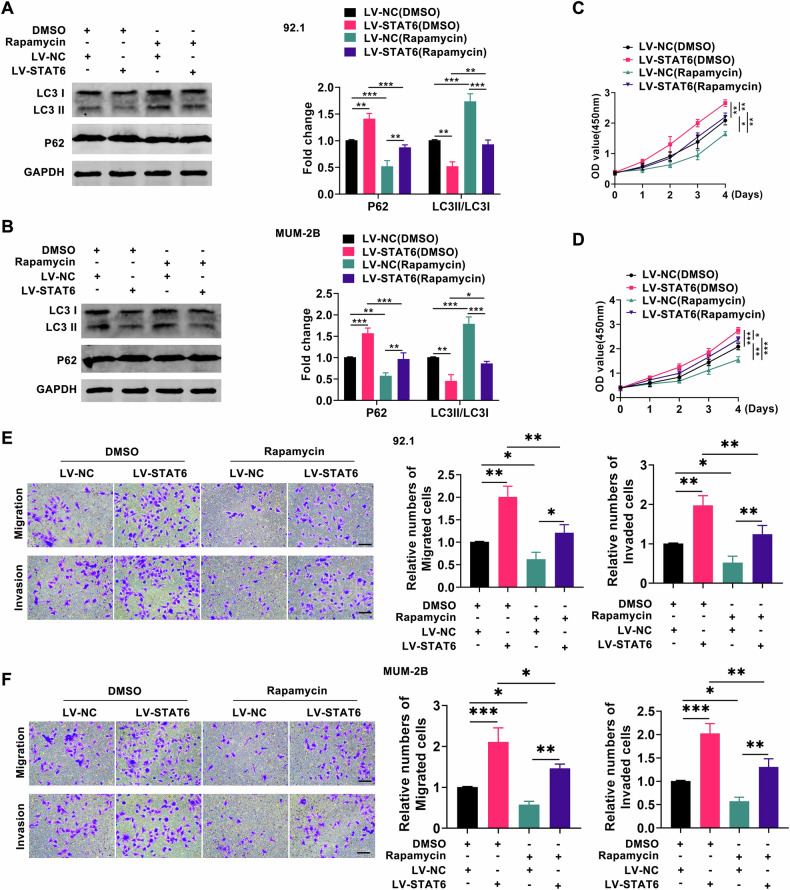
STAT6 promotes the growth of UM cells by inhibiting autophagy. **A**, **B** The expression level of LC3, STAT6, and P62 were analyzed by Western blotting in the LV-NC group and LV-STAT6 group following treatment with either DMSO or rapamycin; (**C**–**F**) The analysis of proliferation, migration, and invasion of the LV-NC group and LV-STAT6 group was conducted following treatment with either DMSO or rapamycin. (Scale bar: 100 µm; Data were presented as the mean ± SD; *n* = 3; *p < 0.05, **p < 0.01, ***p < 0.001).

Previous research has demonstrated that PTK6/SOCS3 influences UM progression via the autophagy pathway, with SOCS3 serving as a key suppressor of the STAT pathway [16]. Therefore, we hypothesized that STAT6 may similarly impact the autophagy pathway in UM. To examine this hypothesis, we evaluated autophagy indicators in UM cells following STAT6 knockdown or overexpression through western blotting experiments. As depicted in Fig. S3A, B, P62 levels decreased and the ratio of LC3 II/LC3 I increased in the knockdown STAT6 group compared to the control group, indicative of autophagy pathway activation. Conversely, as depicted in Fig. S3C, D, P62 levels increased and the ratio of LC3 II/LC3 I decreased in the overexpression of STAT6 group, suggesting inhibition of the autophagy pathway. The above data indicated that STAT6 inhibited the autophagy pathway in UM cells. To investigate whether STAT6 promotes UM growth via the autophagic pathway, we supplemented overexpressed STAT6 or control UM cells with either DMSO or the autophagy agonist rapamycin, based on previous studies, and subsequently assessed the proliferation, migration, and invasion capacities of UM cells. As depicted in Fig. 3A, B, rapamycin mitigated the inhibitory effect of STAT6 on autophagy in UM cells compared to the control. As illustrated in Fig. 3C–F, the proliferation, migration, and invasion capacities of UM cells were reduced in the rapamycin-treated group, particularly evident in the overexpression of STAT6 group. The above data suggested that STAT6 promotes UM progression by inhibiting autophagy.

### LINC01637 binds to STAT6 protein and regulates STAT6 expression

**Fig. 4:**
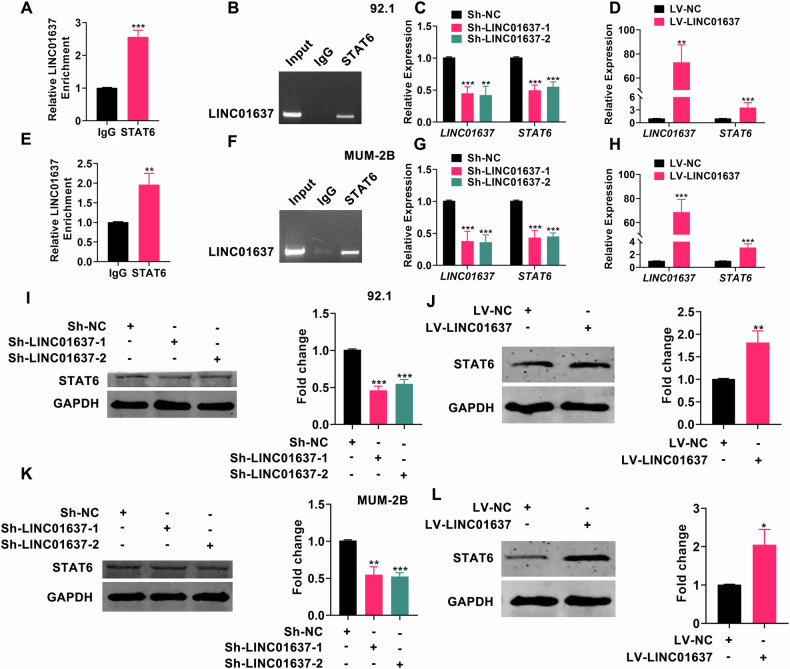
LINC01637 binds to STAT6 protein and regulates STAT6 expression. **A**, **E** Relative enrichment of LINC01637 mRNA associated with STAT6 protein was identified by RIP assays. The IgG group was a negative control to preclude nonspecific binding; (**B**, **F**) Agarose gel electrophoresis results by using RT-qPCR products from RIP assays; (**C**, **G**) The levels of LINC01637 and STAT6 mRNA were measured in UM cells after suppressing LINC01637; (**D**, **H**) The levels of LINC01637 and STAT6 mRNA were measured in UM cells that had an increased expression of LINC01637; (**I**, **K**) STAT6 in UM cells was analyzed by Western blotting, specifically focusing on the cells with LINC01637 knockdown; (**J**, **L**) Results from a Western blotting study of STAT6 in LINC01637-overexpressing UM cells. (Data were presented as the mean ± SD; *n* = 3; *p < 0.05, **p < 0.01, ***p < 0.001).

To delve deeper into the upstream regulatory mechanism of STAT6, we examined its correlation with autophagy-related LNCRNAs as documented in prior studies [23]. As depicted in Fig. S4A, STAT6 exhibited positive correlations with LINC01637, AC036214.2, AP003352.1, and SOX1-OT, and negative correlations with UBXN10-AS1, AC090617.5, and LINC01278. Subsequently, based on the expression profiles and prognostic significance of these seven autophagy-associated LNCRNAs in UM (Fig S4B–H), we identified LINC01637 as the closest association with STAT6. For further elucidation of whether LINC01637 regulates STAT6, we conducted RIP-qPCR experiments to ascertain if STAT6 binds directly to LINC01637. As illustrated in Fig. 4A, B, E, F, relative to the control group (IgG), the STAT6 antibody notably enriched the mRNA of LINC01637, thereby confirming direct binding between STAT6 and LINC01637 mRNA. Subsequently, we induced knockdown or overexpression of LINC01637 in UM cells and assessed the expression levels of STAT6 mRNA and protein. As depicted in Fig. 4C, D, G, H, UM cells exhibited effective knockdown or overexpression of LINC01637, resulting in decreased and increased mRNA expression of STAT6, respectively. Moreover, the protein expression of STAT6 following knockdown or overexpression of LINC01637 corresponded to the aforementioned changes (Fig. 4I–L). These data collectively demonstrated the binding of STAT6 to LINC01637 and the positive regulatory role of LINC01637 in STAT6 expression.

### LINC01637 promotes the progression of UM in vivo and in vitro

**Fig. 5:**
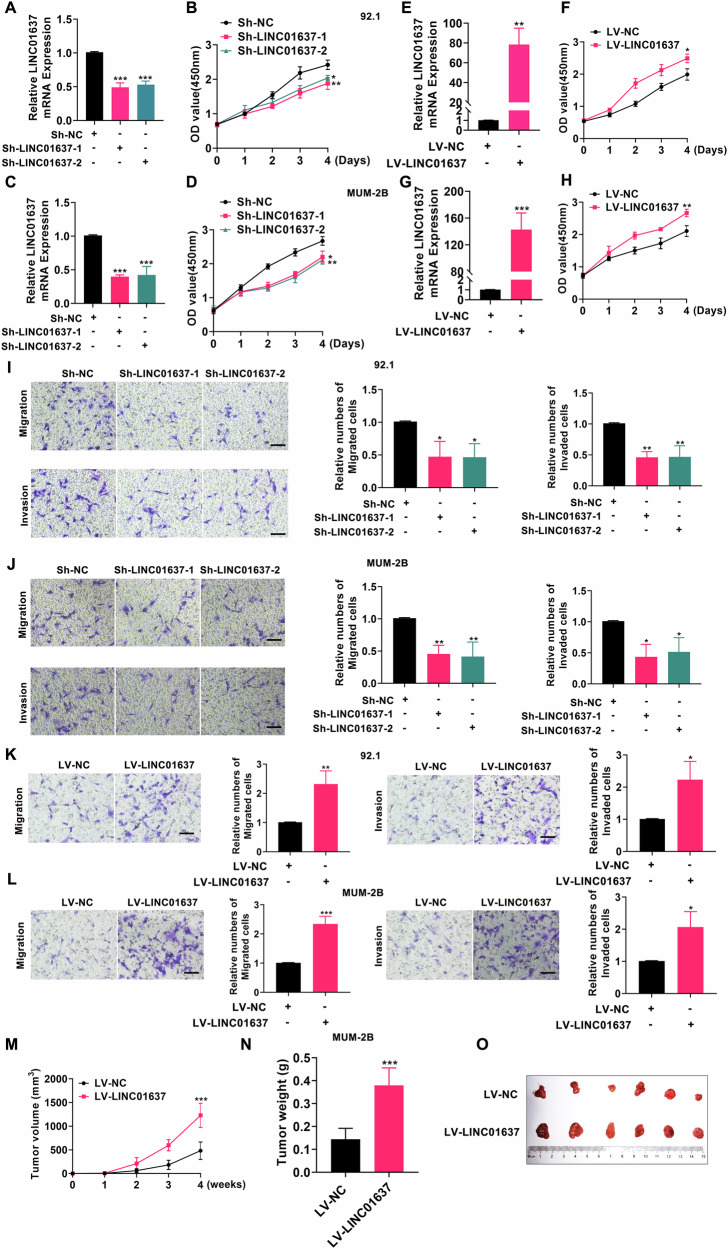
LINC01637 promotes the progression of UM in vivo and in vitro. **A**, **C** The level of LINC01637 mRNA in UM cells with reduced expression of LINC01637; (**B**, **D**) Analysis of the proliferation of UM cells with LINC01637 knockdown by CCK8 assay; (**E**, **G**) The level of LINC01637 mRNA in UM cells that have an increased amount of LINC01637; (**F**, **H**) Analysis of the proliferation of UM cells overexpressing LINC01637 by CCK8 assay; (**I**, **J**) Examination of the migration and invasion of UM cells under LINC01637 knockdown; (**K**, **L**) Examination of the migration and invasion of UM cells overexpressing LINC01637; (**M**) The volume of tumors formed in the LV-NC group and LV-LINC01637 group; (**N**) The weight of tumors formed in the LV-NC group and LV-LINC01637 group; (**O**) Photographic images of tumors from the LV-NC group and LV-LINC01637 group; (Scale bar: 100 µm; Data were presented as the mean ± SD; *n* = 3–6; *p < 0.05, **p < 0.01, ***p < 0.001).

To further elucidate the role of LINC01637 in UM, we assessed its impact on UM cell proliferation, migration, and invasion following knockdown or overexpression. As illustrated in Fig. 5A, C, E, and G, UM cells exhibited successful knockdown or overexpression of LINC01637. Analysis via CCK8 assay revealed that knockdown of LINC01637 inhibited UM cell proliferation (Fig. 5B, D), while overexpression of LINC01637 promoted proliferation (Fig. 5F, H). Concurrently, as depicted in Fig. 5I, J, knockdown of LINC01637 reduced UM cell migration and invasion compared to the control group, whereas overexpression of LINC01637 enhanced these abilities. Lastly, UM cells from the LINC01637 overexpression group and the control group were injected subcutaneously into nude mice. Subsequent monitoring assessed the volume and weight of resulting subcutaneous tumors. As illustrated in Fig. 5M–O and Fig. S5, the volume and weight of subcutaneous tumors were higher in the LINC01637 overexpression group compared to the control group. In summary, LINC01637 promoted the growth of UM in vivo and in vitro.

### LINC01637 promotes the growth of UM by regulating STAT6

**Fig. 6:**
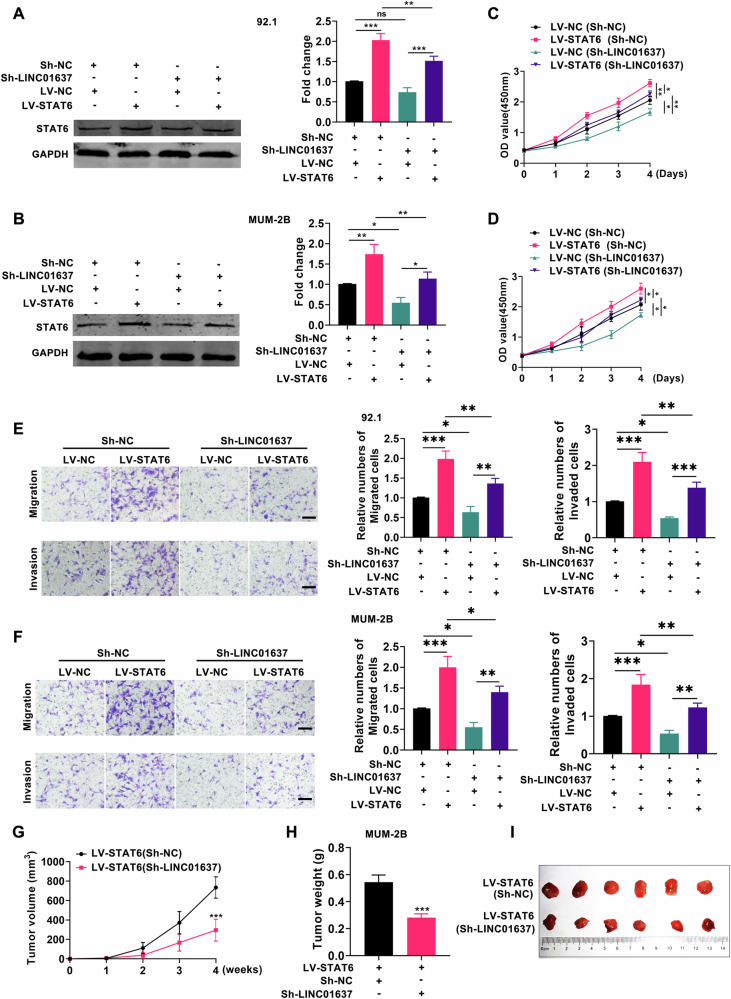
LINC01637 promotes the growth of UM by regulating STAT6. **A**, **B** Western blot analysis was performed to examine the expression of STAT6 in the LV-NC group and LV-STAT6 group, which were supplemented with Sh-NC and Sh-LINC01637; (**C**–**F**) An investigation was conducted to examine the proliferation, migration, and invasion of two groups: the LV-NC group and the LV-STAT6 group. Both groups were supplemented with Sh-NC or Sh-LINC01637; (**G**) The volume of tumors formed in the LV-STAT6/Sh-NC group and LV-STAT6/Sh-LINC01637 group; (**H**) The weight of tumors formed in the LV-STAT6/Sh-NC group and LV-STAT6/Sh-LINC01637 group; (**I**) Photographs of tumors from the LV-STAT6/Sh-NC group and LV-STAT6/Sh-LINC01637 group. (Scale bar: 100 µm; Data were presented as the mean ± SD; *n* = 3–6; *p < 0.05, **p < 0.01, ***p < 0.001).

The aforementioned data suggested that LINC01637 could regulate STAT6 expression. However, whether LINC01637 promotes UM progression through STAT6 remains unclear. Therefore, we conducted further experiments by knocking down LINC01637 in cells already overexpressing STAT6 and subsequently evaluated UM cell proliferation, migration, and invasion. As depicted in Fig. 6A, B, UM cells were successfully transfected with both Sh-NC and Sh-LINC01637 plasmids in conjunction with LV-NC or LV-STAT6. Compared to the control group, UM cell proliferation, migration, and invasion abilities were reduced following additional knockdown of LINC01637 in cells already overexpressing STAT6. Concurrently, cell lines from the LV-STAT6/Sh-NC and LV-STAT6/Sh-LINC01637 groups were injected subcutaneously into nude mice, and subsequent monitoring assessed tumor volume and weight. As depicted in Fig. 6G–I and Fig. S6, tumor volume and weight were reduced in the LV-STAT6/Sh-LINC01637 group compared to the control group. The above data demonstrated that LINC01637 promoted UM progression through STAT6.

### STAT6 is the drug target of Zoledronic Acid

**Fig. 7:**
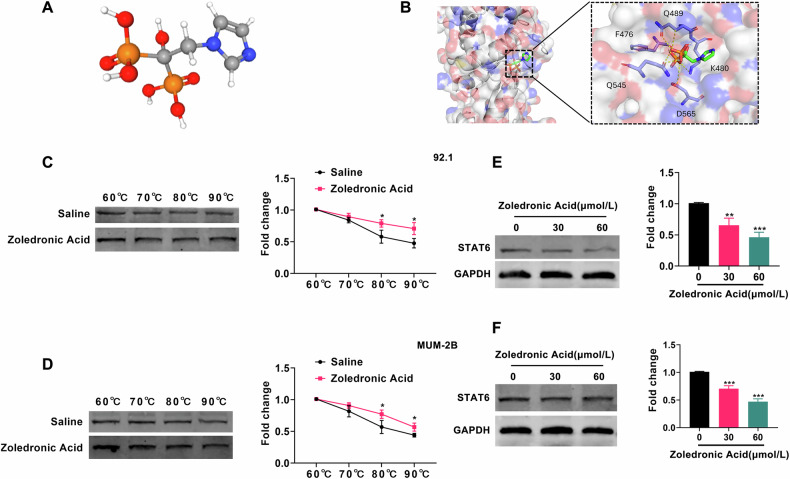
STAT6 is the drug target of Zoledronic Acid. **A** The 3D structural diagram of Zoledronic Acid; (**B**) The molecular docking simulations of STAT6 with Zoledronic Acid; (**C**, **D**) The detection of STAT6 expression in UM cells after saline or Zoledronic Acid treatment at different temperature; (**E**, **F**) The detection of STAT6 expression in UM cells after Zoledronic Acid treatment. (Data were presented as the mean ± SD; *n* = 3; *p < 0.05, **p < 0.01, ***p < 0.001).

For further analysis of the pharmacological role of STAT6 in UM, literature review suggests that STAT6 might serve as a potential drug target for Zoledronic Acid [26]. To validate this hypothesis, we conducted molecular docking modeling to predict the binding interaction between Zoledronic Acid and STAT6. As illustrated in Fig. 7A, B, the prediction results indicated that Zoledronic Acid could indeed bind to the STAT6 protein. Subsequently, we assessed the binding efficiency of the UM intracellular drug Zoledronic Acid to the target protein STAT6 using CETSA (Cellular Thermal Shift Assay). This assay relies on the principle that target proteins stabilize upon binding to drug molecules. As depicted in Fig. 7C, D, the degradation rate of STAT6 protein decreased in UM cells upon addition of Zoledronic Acid compared to the control group (saline). Meanwhile, we incubated STAT6 proteins with biotin-Zoledronic Acid beads in the absence or presence of Zoledronic Acid for pull-down experiments. As shown in Fig. S7, the biotin-Zoledronic Acid beads successfully enriched STAT6 protein compared to the control group, indicating the successful binding of Zoledronic Acid to STAT6 protein. Finally, we treated UM cells with varying concentrations of Zoledronic Acid to assess its impact on STAT6 protein and LINC01637 expression. As depicted in Figs. 7E, F and S8, STAT6 and LINC01637 expression gradually decreased with increasing concentrations of Zoledronic Acid. These data suggest that STAT6 is a drug target of Zoledronic Acid, which inhibits the STAT6-LINC01637 complex.

### Zoledronic Acid inhibits the progression of UM via STAT6 pathway

**Fig. 8:**
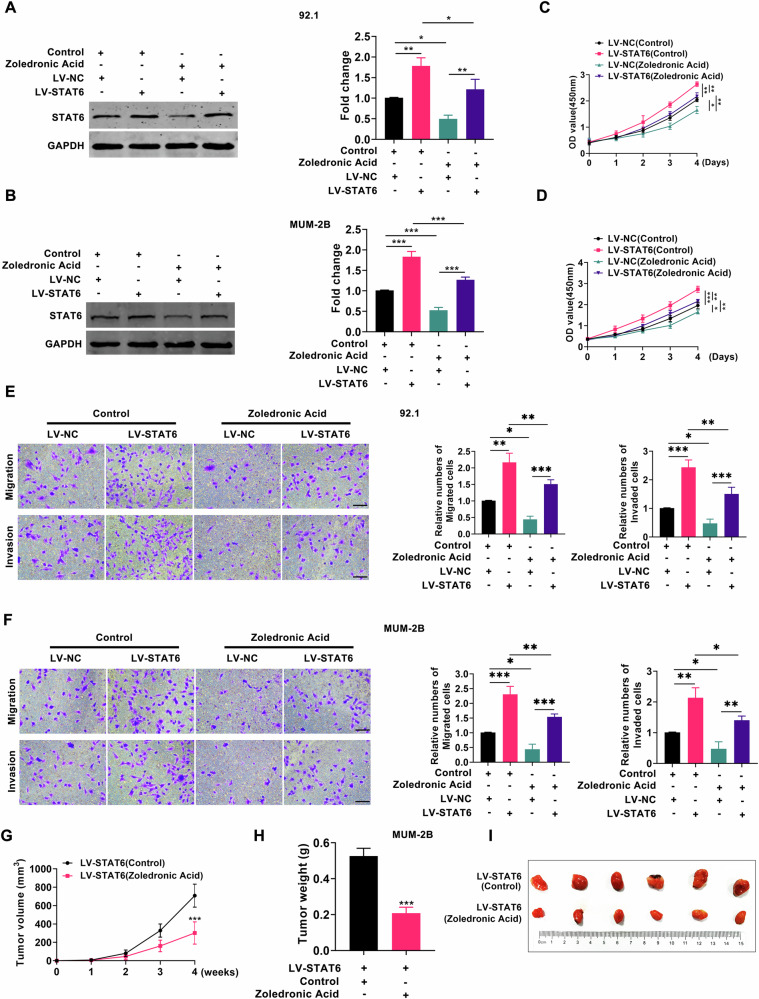
Zoledronic Acid inhibits the progression of UM via STAT6 pathway. **A**, **B** Western blot analysis was conducted to examine the expression of STAT6 in the LV-NC group and LV-STAT6 group following treatment with either saline or Zoledronic Acid; (**C**–**F**) An investigation was conducted to assess the proliferation, migration, and invasion in the LV-NC group and LV-STAT6 group following treatment with either saline or Zoledronic Acid; (**G**) The volume of tumors formed in the LV-STAT6/Control group and LV-STAT6/Zoledronic Acid group; (**H**) The weight of tumors formed in the LV-STAT6/Control group and LV-STAT6/Zoledronic Acid group; (**I**) Photographs of tumors from the LV-STAT6/Control group and LV-STAT6/Zoledronic Acid group. (Control: saline; Scale bar: 100 µm; Data were presented as the mean ± SD; *n* = 3–6; *p < 0.05, **p < 0.01, ***p < 0.001).

We conducted further analysis to investigate the effect of Zoledronic Acid on UM cells. UM cells were treated with various concentrations of Zoledronic Acid, and their proliferation, migration, and invasion abilities were assessed. As depicted in Fig. S9A–D, the proliferation, migration, and invasion abilities of UM cells were reduced upon treatment with Zoledronic Acid compared to the control group. The most significant effect was observed at a concentration of 60 μmol/L. The above data indicated that Zoledronic Acid could inhibit the growth of UM cells. Finally, we would like to investigate whether Zoledronic Acid inhibits UM progression through modulation of the target protein STAT6. Building on the above experimental findings, we treated UM cells in the LV-NC and LV-STAT6 groups with either 60 μmol/L Zoledronic Acid or saline, respectively, and assessed tumor cell proliferation, migration, and invasion. As depicted in Fig. 8A, B, Zoledronic Acid effectively suppressed STAT6 protein expression in both the LV-NC and LV-STAT6 groups compared to the control group. Moreover, supplementation of Zoledronic Acid on the basis of STAT6 overexpression inhibited UM cell proliferation, migration, and invasion (Fig. 8C–F). To further explore the impact of Zoledronic Acid on UM growth through STAT6 in vivo, we subcutaneously injected UM cells overexpressing STAT6 into nude mice and administered daily oral gavage of either saline or 40 mg/kg Zoledronic Acid to the nude mice. As illustrated in Fig. 8G–I and Fig. S10, tumor volume and weight were reduced in the LV-STAT6/Zoledronic Acid group compared to the control group. The above data indicated that Zoledronic Acid inhibited the tumorigenicity of UM by suppressing STAT6.

## Discussion

Long non-coding RNAs (lncRNAs), which are defined as non-protein-coding RNAs spanning more than 200 nucleotides, have emerged as pivotal regulators of gene expression. They are implicated in various biological processes, encompassing cell development, differentiation, and disease progression, particularly in the context of cancer [27]. Extensive research has explored the role of lncRNAs in tumorigenesis and cancer progression, linking their dysregulation to numerous malignancies, among them UM. In the realm of cancer, lncRNAs can act as either oncogenes or tumor suppressors. Oncogenic lncRNAs stimulate tumor growth, invasion, and metastasis, while tumor suppressor lncRNAs impede these processes. Dysregulation of lncRNAs can result in genomic instability, altered cellular metabolism, and resistance to apoptosis, all of which contribute to tumor development and progression [28]. UM, the predominant primary intraocular malignancy among adults, frequently presents high metastatic propensity, thereby entailing a dismal prognosis [4]. Recent studies have discerned particular lncRNAs exhibiting differential expression in UM. For example, the lncRNA MALAT1 stimulates UM cell proliferation and migration through regulation of the miR-608/HOXC4 axis [29]. Another notable lncRNA, PVT1, facilitates the EMT in UM, a pivotal step for tumor invasion and metastasis [30]. Long non-coding RNAs (lncRNAs) show potential as diagnostic and prognostic biomarkers. Their expression profiles can signify tumor stage, aggressiveness, and treatment response. For instance, elevated expression levels of the lncRNA GAS5 correlate with improved survival outcomes in UM patients, indicating its potential as a prognostic indicator [31]. As depicted in Figs. 5 and S4, LINC01637 holds prognostic significance in UM patients; notably, those exhibiting heightened LINC01637 expression display an unfavorable prognosis. Moreover, our findings illustrate that LINC01637 promotes UM progression in both in vivo and in vitro settings. Long non-coding RNAs (lncRNAs) exert their effects through various mechanisms. They may serve as molecular scaffolds, facilitating the assembly of protein complexes, or as decoys, sequestering transcription factors and regulatory proteins [32]. Furthermore, lncRNAs can interact with chromatin to modulate gene expression by altering histones or DNA methylation patterns, thereby impacting gene transcription. They additionally participate in post-transcriptional regulation by influencing mRNA splicing, stability, and translation [33]. In this study, LINC01637 functioned as a molecular sponge for STAT6 proteins, thereby modulating STAT6 activity and influencing the tumorigenicity of UM. Despite significant advances in understanding the role of lncRNAs in cancer, substantial challenges remain. The functional characterization of lncRNAs is complex due to their diverse mechanisms of action and interactions with various cellular components. Moreover, developing targeted therapies necessitates a profound understanding of lncRNA biology and the identification of safe and effective delivery systems. In conclusion, lncRNAs play a pivotal role in the pathogenesis of cancer, including UM. Their multifaceted roles in regulating gene expression and cellular processes render them attractive targets for cancer research and therapy.

STAT6, a signal transducer and activator of transcription, is a critical mediator of cytokine signaling, particularly involved in regulating immune responses, inflammation, and cell growth. STAT6 is activated by the Janus kinase (JAK) family of proteins in response to cytokines, such as IL-4 and IL-13 [34]. Upon activation, STAT6 dimerizes and translocates to the nucleus, where it modulates the expression of genes involved in immune regulation and cell differentiation. Aberrant activation of the JAK-STAT6 pathway has been linked to the pathogenesis of allergic disorders and certain cancers [35]. Developing STAT6 inhibitors has been a focal point in drug discovery efforts. These inhibitors can be broadly classified into small-molecule inhibitors, monoclonal antibodies, and RNA interference-based therapies [35]. Small-Molecule Inhibitors: These compounds target the catalytic activity of JAK proteins, thereby indirectly inhibiting STAT6 phosphorylation and activation. Examples include ruxolitinib, approved for the treatment of myelofibrosis [36], and tofacitinib, used in rheumatoid arthritis [37]. Although these drugs target JAK proteins, they can also affect STAT6 signaling. Monoclonal Antibodies: These are designed to neutralize specific cytokines that activate STAT6. For instance, dupilumab, a monoclonal antibody against the IL-4 receptor alpha subunit, effectively blocks IL-4 and IL-13 signaling, thus inhibiting STAT6 activation. Dupilumab is approved for the treatment of moderate-to-severe atopic dermatitis and asthma [38, 39]. RNA Interference: This approach involves using small interfering RNA (siRNA) or small hairpin RNA (shRNA) to specifically silence STAT6 expression. As shown in Figs. 7, 8, and S9, we found that the target of Zoledronic Acid is STAT6, which can inhibit the growth of UM by regulating the expression of STAT6. Zoledronic Acid is a third-generation aminobisphosphonate that exhibits high affinity for bone mineral, where it is selectively concentrated and taken up by osteoclasts. Zoledronic Acid has been shown to significantly increase bone mineral density and reduce the risk of fractures in patients with osteoporosis [40]. Beyond its effects on bone, Zoledronic Acid has been found to have direct anti-tumor effects. It can induce apoptosis in certain cancer cells and inhibit angiogenesis, which are important for tumor growth and metastasis [41]. Zoledronic Acid has been shown to modulate immune responses, with potential implications for cancer immunotherapy. It can enhance the activity of immune cells against tumor cells and promote the release of cytokines that contribute to an anti-tumor immune response [42]. In this study, we reported for the first time the function and molecular mechanism of Zoledronic Acid in UM, providing valuable insights for future therapeutic strategies for UM.

The complex regulatory network within cells encompasses numerous factors, including proteins, transcription factors, and non-coding RNAs such as long non-coding RNAs (lncRNAs). STAT6, a transcription factor, plays a crucial role in immune responses and is tightly regulated at multiple levels, including by lncRNAs [43]. Certain lncRNAs can directly bind to STAT6, affecting its phosphorylation, dimerization, or nuclear translocation. For example, specific lncRNAs may sequester STAT6 in the cytoplasm, preventing its nuclear entry and subsequent gene regulation [44]. LncRNAs can also modulate the epigenetic landscape surrounding STAT6 target genes, affecting their accessibility and transcriptional activity. This modulation may involve the recruitment of histone-modifying enzymes or chromatin remodeling complexes [45]. LncRNAs may function as transcriptional regulators by affecting STAT6 binding to its cognate DNA elements. They may promote or inhibit the recruitment of STAT6 to specific gene promoters or enhancers. LncRNAs are implicated in the post-transcriptional regulation of STAT6 activity, potentially influencing mRNA splicing, stability, or translation efficiency [28]. Dysregulation of the STAT6-lncRNA axis is implicated in various diseases, especially those involving immune dysregulation. In allergic diseases, STAT6 overactivation can lead to heightened Th2 responses, with specific lncRNAs potentially contributing to this process by enhancing STAT6 activity [46]. Conversely, in cancer, the STAT6-lncRNA interplay may influence tumor growth and immune evasion, thereby impacting cancer progression and therapy resistance [19]. In conclusion, the regulatory interplay between STAT6 and lncRNAs constitutes a complex and nuanced aspect of cellular regulation, with significant implications for immune function and disease pathogenesis. Elucidating the intricacies of this relationship is essential for advancing our understanding of immune signaling and developing novel therapeutic strategies. Previous studies have demonstrated that lncRNAs can predict the prognosis of UM patients [23]. However, the specific regulatory mechanisms remain unknown. In this study, we found that LINC01637 was positively correlated with STAT6 through bioinformatics analysis, suggesting a potential regulatory relationship between them. We discovered that STAT6 protein could bind to LINC01637 mRNA via RIP-PCR, and LINC01637 promoted UM progression by positively regulating STAT6. This suggests that the STAT6/LINC01637 axis is involved in the tumorigenicity of UM and provides a new research target for future molecular therapy of UM.

In summary, we identified that STAT family genes have prognostic significance in UM patients. STAT6 promotes UM progression both in vivo and in vitro via the autophagy pathway. Zoledronic Acid targets STAT6 and delays UM tumorigenicity by inhibiting its expression. Mechanistically, STAT6 binds to LINC01637 mRNA, and LINC01637 regulates STAT6 expression. Our work demonstrates the role of the STAT6/LINC01637 axis in UM, providing novel insights for clinical treatment.

## Supplementary information

Supplementary materials

Original Data

## Data Availability

The datasets generated or analyzed in this study are available upon reasonable request to the corresponding author.

## References

[1] Carvajal RD, Sacco JJ, Jager MJ, Eschelman DJ, Olofsson Bagge R, Harbour JW, et al. Advances in the clinical management of uveal melanoma. Nat Rev Clin Oncol. 2023;20:99–115. 36600005 10.1038/s41571-022-00714-1

[2] Rantala ES, Hernberg MM, Piperno-Neumann S, Grossniklaus HE, Kivelä TT. Metastatic uveal melanoma: the final frontier. Prog Retin Eye Res. 2022;90:101041. 34999237 10.1016/j.preteyeres.2022.101041

[3] Liu B, Yao X, Shang Y, Dai J. The multiple roles of autophagy in uveal melanoma and the microenvironment. J Cancer Res Clin Oncol. 2024;150:121. 38467935 10.1007/s00432-023-05576-3 PMC10927889

[4] Wespiser M, Neidhardt E, Negrier S. Uveal melanoma: in the era of new treatments. Cancer Treat Rev. 2023;119:102599. 37473516 10.1016/j.ctrv.2023.102599

[5] Yeşiltaş YS, Oakey Z, Wrenn J, Yeaney G, Brainard J, Lorek B, et al. Uveal melanoma in African Americans: diagnostic challenges. Surv Ophthalmol. 2024;69:190–7. 37406779 10.1016/j.survophthal.2023.06.011

[6] Khan S, Carvajal RD. Dual immunological checkpoint blockade for Uveal Melanoma. J Clin Oncol. 2021;39:554–6. 33417487 10.1200/JCO.20.03274

[7] Lietman CD, McKean M. Targeting GNAQ/11 through PKC inhibition in uveal melanoma. Cancer Gene Ther. 2022;29:1809–13. 35181742 10.1038/s41417-022-00437-6

[8] Hu X, Li J, Fu M, Zhao X, Wang W. The JAK/STAT signaling pathway: from bench to clinic. Signal Transduct Target Ther. 2021;6:402. 34824210 10.1038/s41392-021-00791-1 PMC8617206

[9] Li Y-J, Zhang C, Martincuks A, Herrmann A, Yu H. STAT proteins in cancer: orchestration of metabolism. Nat Rev Cancer. 2023;23:115–34. 36596870 10.1038/s41568-022-00537-3

[10] Avalle L, Raggi L, Monteleone E, Savino A, Viavattene D, Statello L, et al. STAT3 induces breast cancer growth via ANGPTL4, MMP13 and STC1 secretion by cancer associated fibroblasts. Oncogene. 2022;41:1456–67. 35042959 10.1038/s41388-021-02172-y

[11] Ding L, Wang Q, Martincuks A, Kearns MJ, Jiang T, Lin Z, et al. STING agonism overcomes STAT3-mediated immunosuppression and adaptive resistance to PARP inhibition in ovarian cancer. J Immunother Cancer. 2023;11:e005627. 36609487 10.1136/jitc-2022-005627 PMC9827255

[12] Moreira D, Sampath S, Won H, White SV, Su Y-L, Alcantara M, et al. Myeloid cell-targeted STAT3 inhibition sensitizes head and neck cancers to radiotherapy and T cell-mediated immunity. J Clin Investig. 2021;131:e137001. 33232304 10.1172/JCI137001 PMC7810478

[13] Zou S, Tong Q, Liu B, Huang W, Tian Y, Fu X. Targeting STAT3 in cancer immunotherapy. Mol Cancer. 2020;19:145. 32972405 10.1186/s12943-020-01258-7 PMC7513516

[14] Butturini E, Carcereri de Prati A, Mariotto S. Redox regulation of STAT1 and STAT3 signaling. Int J Mol Sci. 2020;21:7034. 32987855 10.3390/ijms21197034 PMC7582491

[15] Yang C, Mai H, Peng J, Zhou B, Hou J, Jiang D. STAT4: an immunoregulator contributing to diverse human diseases. Int J Biol Sci. 2020;16:1575–85. 32226303 10.7150/ijbs.41852 PMC7097918

[16] Liu B, Yao X, Zhang C, Liu Y, Wei L, Huang Q, et al. PTK6 inhibits autophagy to promote uveal melanoma tumorigenesis by binding to SOCS3 and regulating mTOR phosphorylation. Cell Death Dis. 2023;14:55. 36690663 10.1038/s41419-023-05590-w PMC9870980

[17] Stark JM, Tibbitt CA, Coquet JM. The metabolic requirements of Th2 cell differentiation. Front Immunol. 2019;10:2318. 31611881 10.3389/fimmu.2019.02318 PMC6776632

[18] Soliman E, Leonard J, Basso EKG, Gershenson I, Ju J, Mills J, et al. Efferocytosis is restricted by axon guidance molecule EphA4 via ERK/Stat6/MERTK signaling following brain injury. J Neuroinflamm. 2023;20:256. 10.1186/s12974-023-02940-5 PMC10633953 37941008

[19] Yu D, Zhao Z, Wang L, Qiao S, Yang Z, Wen Q, et al. SOX21-AS1 activated by STAT6 promotes pancreatic cancer progression via up-regulation of SOX21. J Transl Med. 2022;20:511. 36335356 10.1186/s12967-022-03521-5 PMC9636668

[20] Kamerkar S, Leng C, Burenkova O, Jang SC, McCoy C, Zhang K, et al. Exosome-mediated genetic reprogramming of tumor-associated macrophages by exoASO-STAT6 leads to potent monotherapy antitumor activity. Sci Adv. 2022;8:eabj7002. 35179953 10.1126/sciadv.abj7002 PMC8856615

[21] Wan B, Liu B, Yu G, Huang Y, Lv C. Differentially expressed autophagy-related genes are potential prognostic and diagnostic biomarkers in clear-cell renal cell carcinoma. Aging. 2019;11:9025–42. 31626592 10.18632/aging.102368 PMC6834403

[22] Xu F, Huang X, Li Y, Chen Y, Lin L. m6A-related lncRNAs are potential biomarkers for predicting prognoses and immune responses in patients with LUAD. Mol Ther Nucleic Acids. 2021;24:780–91. 33996259 10.1016/j.omtn.2021.04.003 PMC8094594

[23] Liu B, Yao X, Zhang C, Li W, Wang Y, Liao Q, et al. LINC01278 induces autophagy to inhibit tumour progression by suppressing the mTOR signalling pathway. Oxid Med Cell Longev. 2023;2023:8994901. 36713034 10.1155/2023/8994901 PMC9876672

[24] Li H, Liu B, Lian L, Zhou J, Xiang S, Zhai Y, et al. High dose expression of heme oxigenase-1 induces retinal degeneration through ER stress-related DDIT3. Mol Neurodegener. 2021;16:16. 33691741 10.1186/s13024-021-00437-4 PMC7944639

[25] Jin Y, Zhang B, Lu J, Song Y, Wang W, Zhang W, et al. Long noncoding RNA PM maintains cerebellar synaptic integrity and Cbln1 activation via Pax6/Mll1-mediated H3K4me3. PLoS Biol. 2021;19:e3001297. 34111112 10.1371/journal.pbio.3001297 PMC8219131

[26] Kuninty PR, Binnemars-Postma K, Jarray A, Pednekar KP, Heinrich MA, Pijffers HJ, et al. Cancer immune therapy using engineered ‛tail-flipping’ nanoliposomes targeting alternatively activated macrophages. Nat Commun. 2022;13:4548. 35927238 10.1038/s41467-022-32091-9 PMC9352736

[27] Yan H, Bu P. Non-coding RNA in cancer. Essays Biochem. 2021;65:625–39. 33860799 10.1042/EBC20200032 PMC8564738

[28] Shaath H, Vishnubalaji R, Elango R, Kardousha A, Islam Z, Qureshi R, et al. Long non-coding RNA and RNA-binding protein interactions in cancer: experimental and machine learning approaches. Semin Cancer Biol. 2022;86:325–45. 35643221 10.1016/j.semcancer.2022.05.013

[29] Wu S, Chen H, Zuo L, Jiang H, Yan H. Suppression of long noncoding RNA MALAT1 inhibits the development of uveal melanoma via microRNA-608-mediated inhibition of HOXC4. Am J Physiol Cell Physiol. 2020;318:C903–12. 31913701 10.1152/ajpcell.00262.2019 PMC7294322

[30] Wu S, Chen H, Han N, Zhang C, Yan H. Long noncoding RNA PVT1 silencing prevents the development of Uveal melanoma by impairing MicroRNA-17-3p-dependent MDM2 upregulation. Investig Ophthalmol Vis Sci. 2019;60:4904–14. 31770435 10.1167/iovs.19-27704

[31] Qi Y, Cui Q, Zhang W, Yao R, Xu D, Zhang F. Long non-coding RNA GAS5 Targeting microRNA-21 to suppress the invasion and epithelial-mesenchymal transition of Uveal Melanoma. Cancer Manag Res. 2020;12:12259–67. 33273862 10.2147/CMAR.S260866 PMC7708682

[32] Ransohoff JD, Wei Y, Khavari PA. The functions and unique features of long intergenic non-coding RNA. Nat Rev Mol Cell Biol. 2018;19:143–57. 29138516 10.1038/nrm.2017.104 PMC5889127

[33] Khorkova O, Stahl J, Joji A, Volmar C-H, Zeier Z, Wahlestedt C. Long non-coding RNA-targeting therapeutics: discovery and development update. Expert Opin Drug Discov. 2023;18:1011–29. 37466388 10.1080/17460441.2023.2236552

[34] STAT6 Gain-of-Function International Consortium. Electronic address: sturvey@bcchr.ca; STAT6 Gain-of-Function International Consortium. Human germline gain-of-function in STAT6: from severe allergic disease to lymphoma and beyond. Trends Immunol. 2024;45:138–53. 38238227 10.1016/j.it.2023.12.003

[35] Wong GL, Manore SG, Doheny DL, Lo H-W. STAT family of transcription factors in breast cancer: pathogenesis and therapeutic opportunities and challenges. Semin Cancer Biol. 2022;86:84–106. 35995341 10.1016/j.semcancer.2022.08.003 PMC9714692

[36] Al-Ali HK, Griesshammer M, Foltz L, Palumbo GA, Martino B, Palandri F, et al. Primary analysis of JUMP, a phase 3b, expanded-access study evaluating the safety and efficacy of ruxolitinib in patients with myelofibrosis, including those with low platelet counts. Br J Haematol. 2020;189:888–903. 32017044 10.1111/bjh.16462

[37] Khosrow-Khavar F, Kim SC, Lee H, Lee SB, Desai RJ. Tofacitinib and risk of cardiovascular outcomes: results from the Safety of TofAcitinib in Routine care patients with Rheumatoid Arthritis (STAR-RA) study. Ann Rheum Dis. 2022;81:798–804. 35027405 10.1136/annrheumdis-2021-221915 PMC9117457

[38] Reich K, Thyssen JP, Blauvelt A, Eyerich K, Soong W, Rice ZP, et al. Efficacy and safety of abrocitinib versus dupilumab in adults with moderate-to-severe atopic dermatitis: a randomised, double-blind, multicentre phase 3 trial. Lancet. 2022;400:273–82. 35871814 10.1016/S0140-6736(22)01199-0

[39] Castro M, Corren J, Pavord ID, Maspero J, Wenzel S, Rabe KF, et al. Dupilumab efficacy and safety in moderate-to-severe uncontrolled asthma. N. Engl J Med. 2018;378:2486–96. 29782217 10.1056/NEJMoa1804092

[40] Wang B, Zhan Y, Yan L, Hao D. How zoledronic acid improves osteoporosis by acting on osteoclasts. Front Pharm. 2022;13:961941. 10.3389/fphar.2022.961941 PMC9452720 36091799

[41] Wang L, Fang D, Xu J, Luo R. Various pathways of zoledronic acid against osteoclasts and bone cancer metastasis: a brief review. BMC Cancer. 2020;20:1059. 33143662 10.1186/s12885-020-07568-9 PMC7607850

[42] Zakeri N, Hall A, Swadling L, Pallett LJ, Schmidt NM, Diniz MO, et al. Characterisation and induction of tissue-resident gamma delta T-cells to target hepatocellular carcinoma. Nat Commun. 2022;13:1372. 35296658 10.1038/s41467-022-29012-1 PMC8927126

[43] Zhang S, Sun W-C, Liang Z-d, Yin X-R, Ji Z-R, Chen X-H, et al. LncRNA SNHG4 attenuates inflammatory responses by sponging miR-449c-5p and up-regulating STAT6 in microglial during cerebral ischemia-reperfusion injury. Drug Des Devel Ther. 2020;14:3683–95. 32982175 10.2147/DDDT.S245445 PMC7494233

[44] Jiang L, Zhao X-H, Mao Y-L, Wang J-F, Zheng H-J, You Q-S. Long non-coding RNA RP11-468E2.5 curtails colorectal cancer cell proliferation and stimulates apoptosis via the JAK/STAT signaling pathway by targeting STAT5 and STAT6. J Exp Clin Cancer Res. 2019;38:465. 31718693 10.1186/s13046-019-1428-0 PMC6852742

[45] Zhou J, Li Z, Wu T, Zhao Q, Zhao Q, Cao Y. LncGBP9/miR-34a axis drives macrophages toward a phenotype conducive for spinal cord injury repair via STAT1/STAT6 and SOCS3. J Neuroinflamm. 2020;17:134. 10.1186/s12974-020-01805-5 PMC7187522 32345320

[46] Hu G, Tang Q, Sharma S, Yu F, Escobar TM, Muljo SA, et al. Expression and regulation of intergenic long noncoding RNAs during T cell development and differentiation. Nat Immunol. 2013;14:1190–8. 24056746 10.1038/ni.2712 PMC3805781

