# Supplementary material for: STAT6/LINC01637 axis regulates tumor growth via autophagy and pharmacological targeting STAT6 as a novel strategy for uveal melanoma
Source: Cell Death Dis. 2024 Oct 1;15(10):713. doi: 10.1038/s41419-024-07115-5 (PMC11445459; doi:10.1038/s41419-024-07115-5)
Supplement: Supplementary file 1 — Supplementary materials [file 41419_2024_7115_MOESM1_ESM.docx]

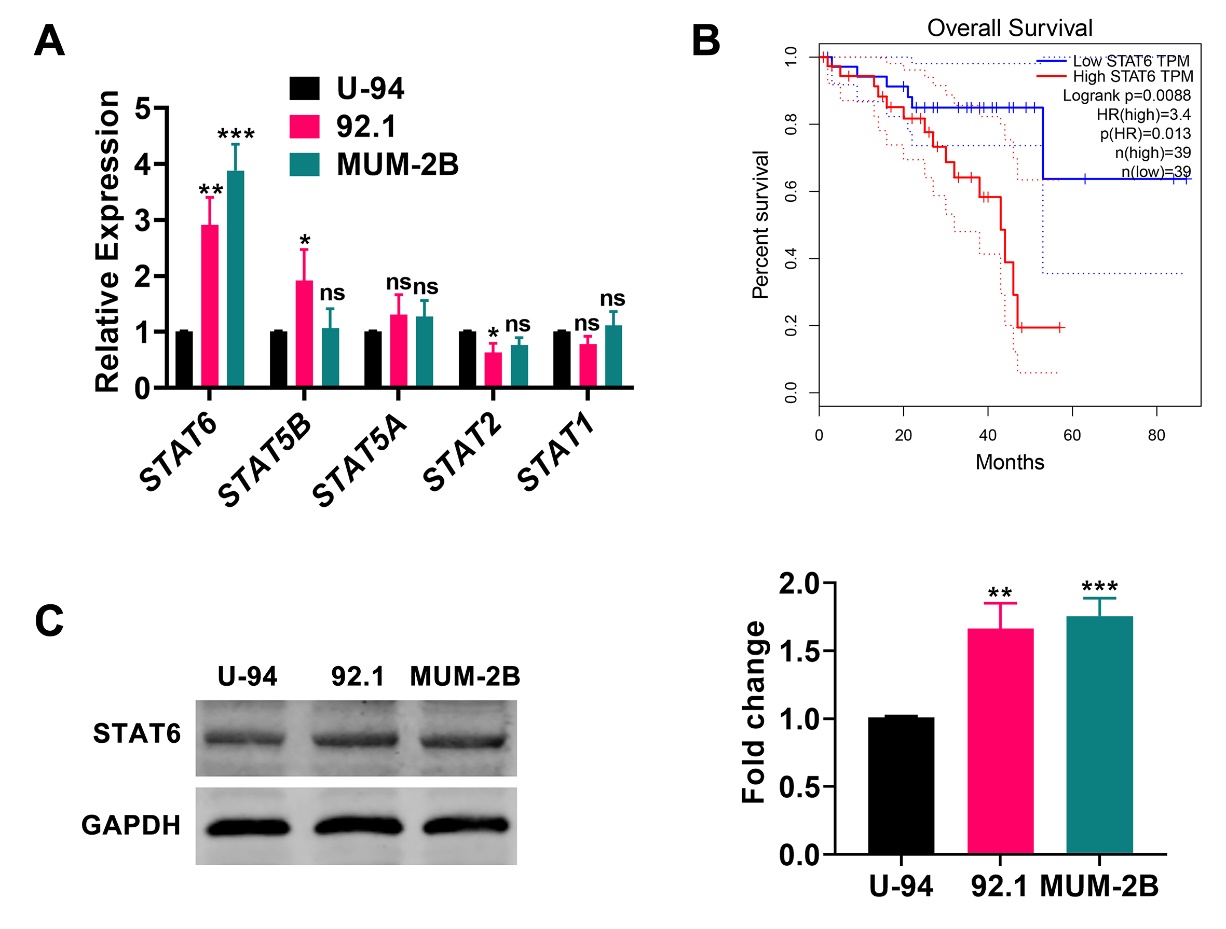


**Figure S1. STAT6 expression is correlated with the survival of UM patients.**

**A:** The expression of 5 STAT family gene mRNAs in U-94 and UM cells; **B:** OS curves for UM patients in the STAT6 high-expression group and the STAT6 low-expression group; **C:** Western blotting analysis of STAT6 in U-94 and UM cells. (Data are presented as the mean ± SD; n = 3; ns: no significant difference, * p < 0.05, ** p < 0.01, *** p < 0.001).


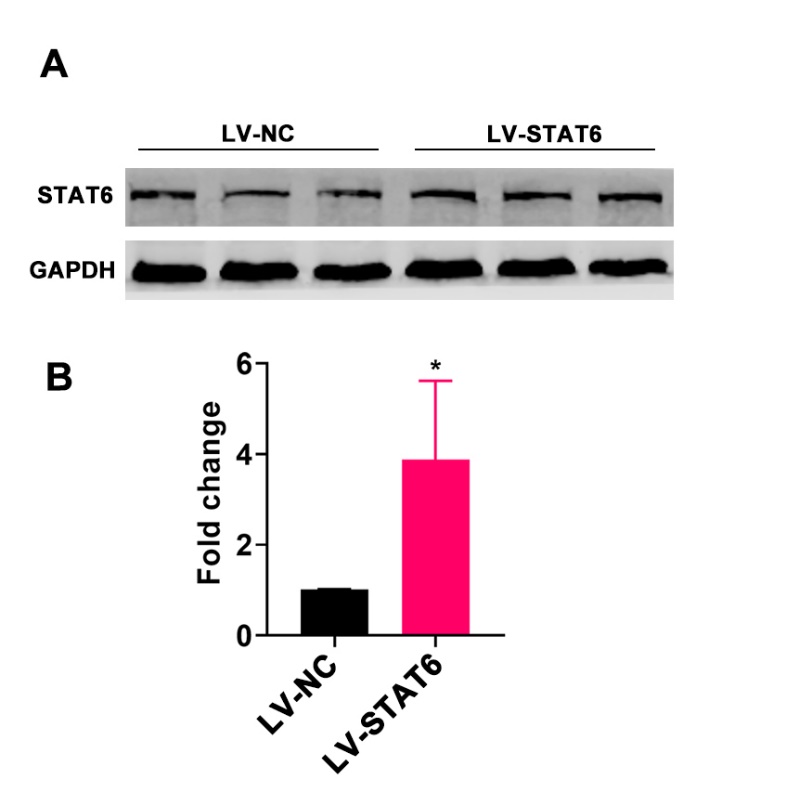


**Figure S2.** **The expression of STAT6 in nude mouse samples.**

**A-B:** Western blotting analysis of STAT6 in LV-NC and LV-STAT6. (Data are presented as the mean ± SD; n = 3; * p < 0.05).


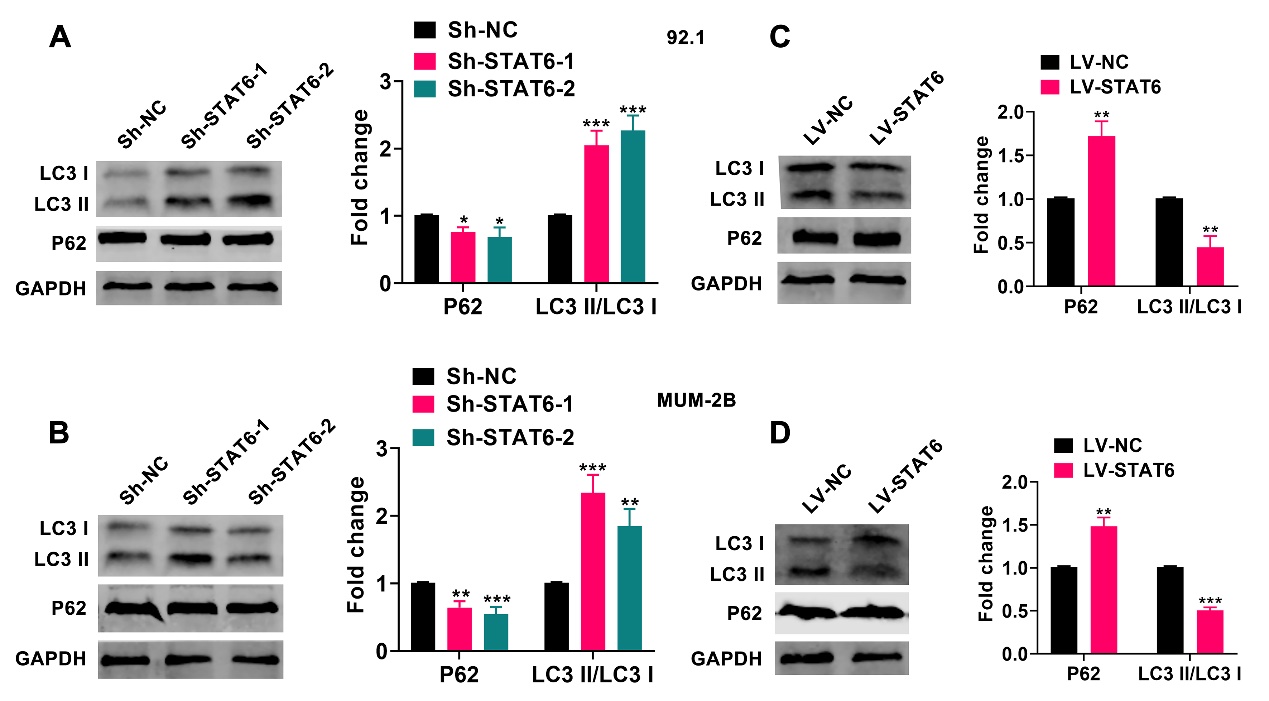


**Figure S3. STAT6 inhibits autophagy in UM cells.**

**A and B:** Western blotting analysis of LC3 and P62 in UM cells with STAT6 knockdown; **C and D:** Western blotting analysis of LC3 and P62 in UM cells overexpressing STAT6. (Data were presented as the mean ± SD; n = 3; * p < 0.05, ** p < 0.01, *** p < 0.001).


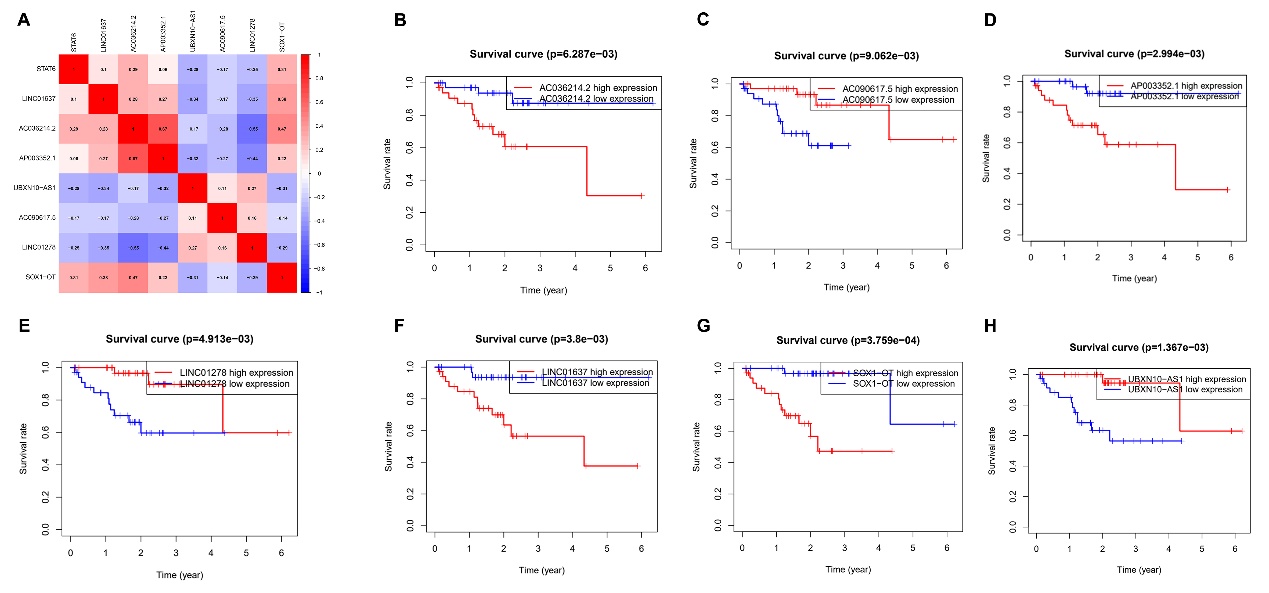


**Figure S4.** **Prognostic value of LINC01637 in UM patients.**

**A:** Correlation of STAT6 and 7 autophagy-related lncRNAs in UM patients; **B-H:** Prognostic analysis of 7 autophagy-related lncRNAs in UM patients.


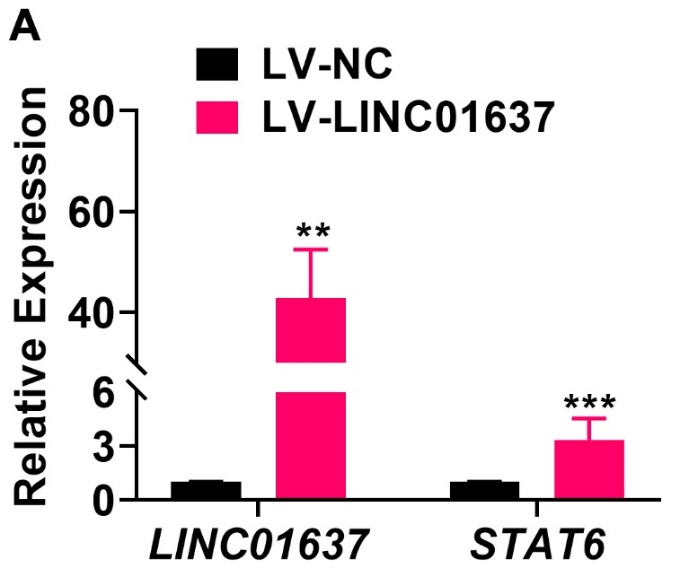


**Figure S5. The expression of *LINC01637* and *STAT6* in nude mouse samples.**

**A:** Q-PCR analysis of the expression of *LINC01637* and *STAT6* in LV-NC and LV-LINC01637. (Data are presented as the mean ± SD; n = 3; ** p < 0.01, *** p < 0.001).


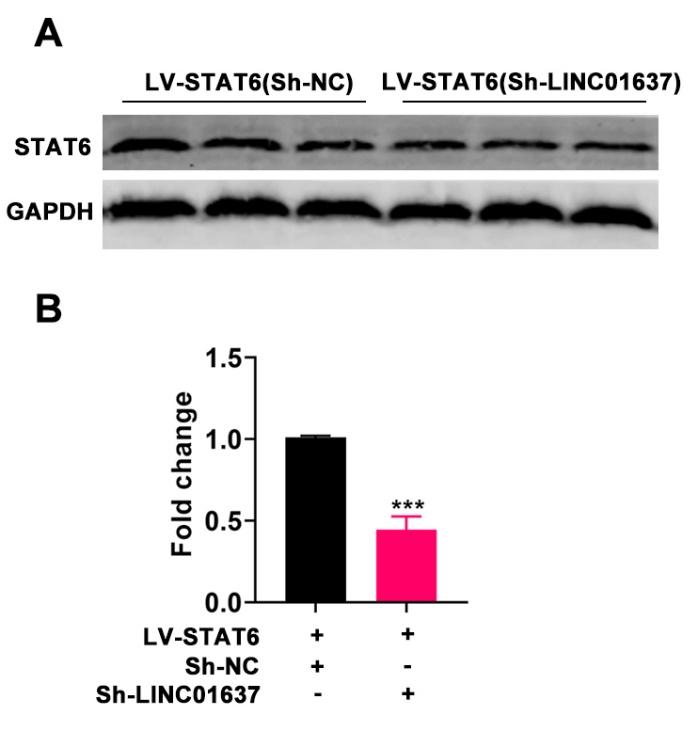


**Figure S6.** **The expression of STAT6 in nude mouse samples.**

**A-B:** Western blotting analysis of STAT6 in LV-STAT6(Sh-NC) and LV-STAT6(Sh-LINC01637). (Data are presented as the mean ± SD; n = 3; *** p < 0.001).


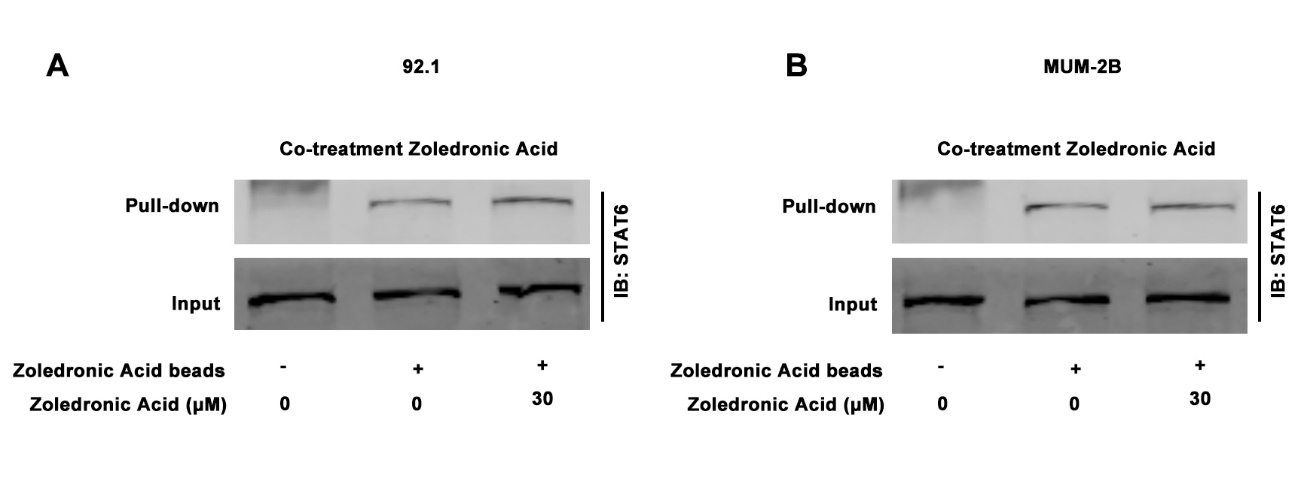


**Figure S7.** **The STAT6 protein can bind to Zoledronic Acid.**

**A-B:** The STAT6 protein was incubated with Biotin-Zoledronic Acid beads in the absence or presence of Zoledronic Acid for binding.


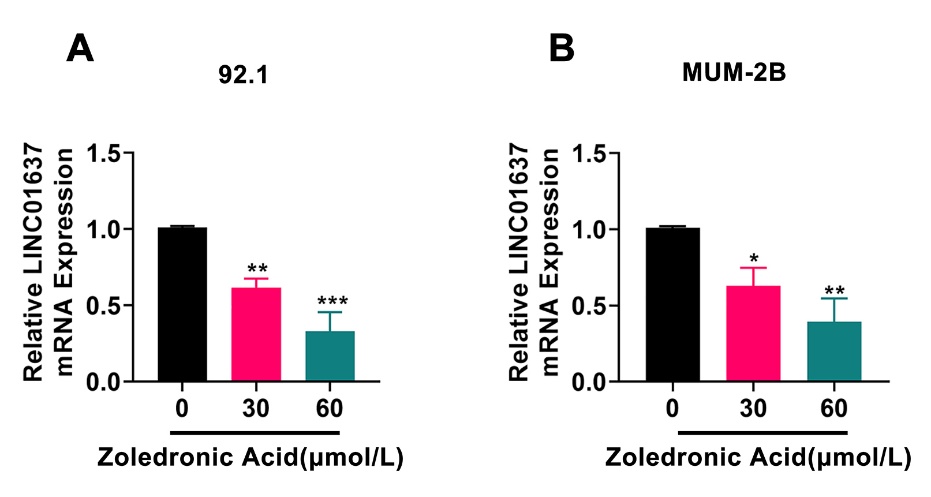


**Figure S8.** **Zoledronic Acid inhibits LINC01637 expression in UM cell lines.**

**A-B.** Q-PCR analysis of the expression of LINC01637 in UM cells after Zoledronic Acid treatment. (Data are presented as the mean ± SD; n = 3; * p < 0.05, ** p < 0.01, *** p < 0.001).


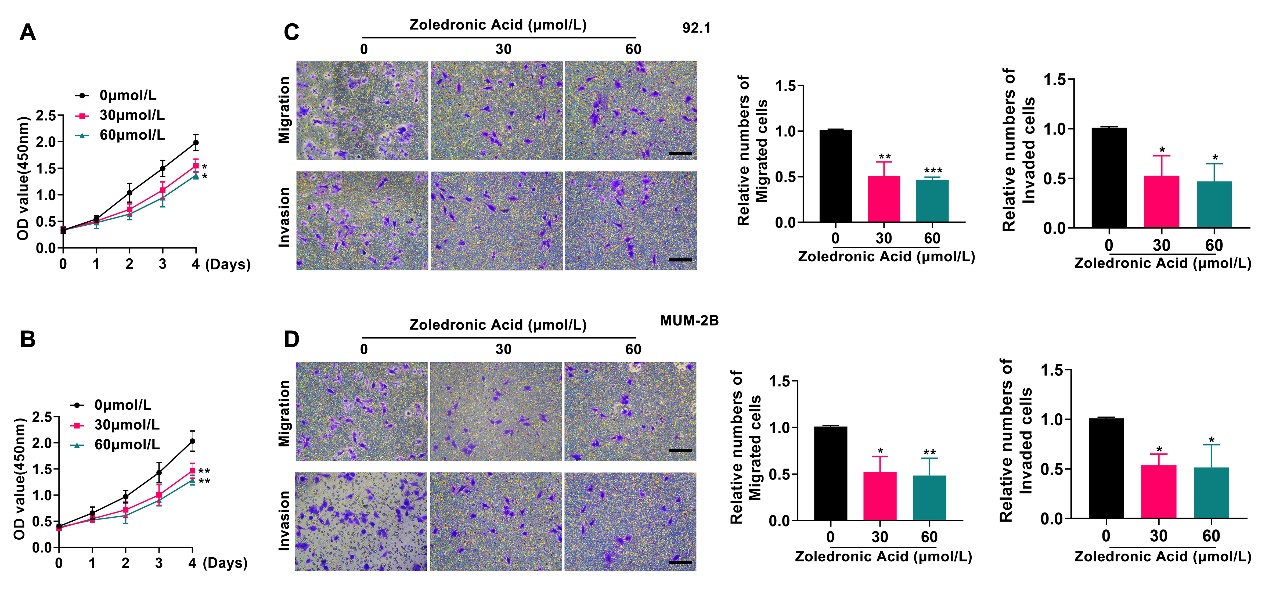


**Figure S9. Zoledronic Acid inhibits the progression of UM in vitro.**

**A and B:** Analysis of the proliferation of UM cell with Zoledronic Acid treatment; **C and D:** Analysis of the migration and invasion of UM cell with Zoledronic Acid treatment. (Scale bar: 100 µm; Data are presented as the mean ± SD; n = 3; * p < 0.05, ** p < 0.01, *** p < 0.001).


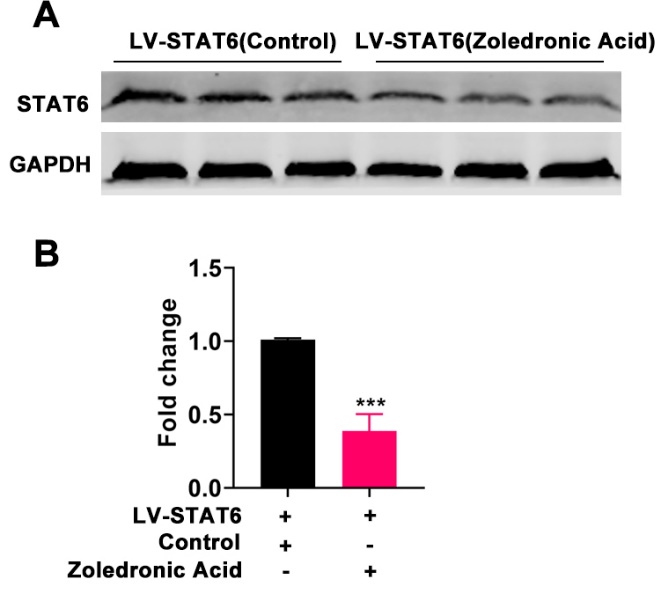


**Figure S10. The expression of STAT6 in nude mouse samples.**

**A-B:** Western blotting analysis of STAT6 in LV-STAT6(Control) and LV-STAT6(Zoledronic Acid). (Data are presented as the mean ± SD; n = 3; *** p < 0.001).
