# Supplementary figures and images for: STAT6/LINC01637 axis regulates tumor growth via autophagy and pharmacological targeting STAT6 as a novel strategy for uveal melanoma
Source: Cell Death Dis. 2024 Oct 1;15(10):713. doi: 10.1038/s41419-024-07115-5 (PMC11445459; doi:10.1038/s41419-024-07115-5)

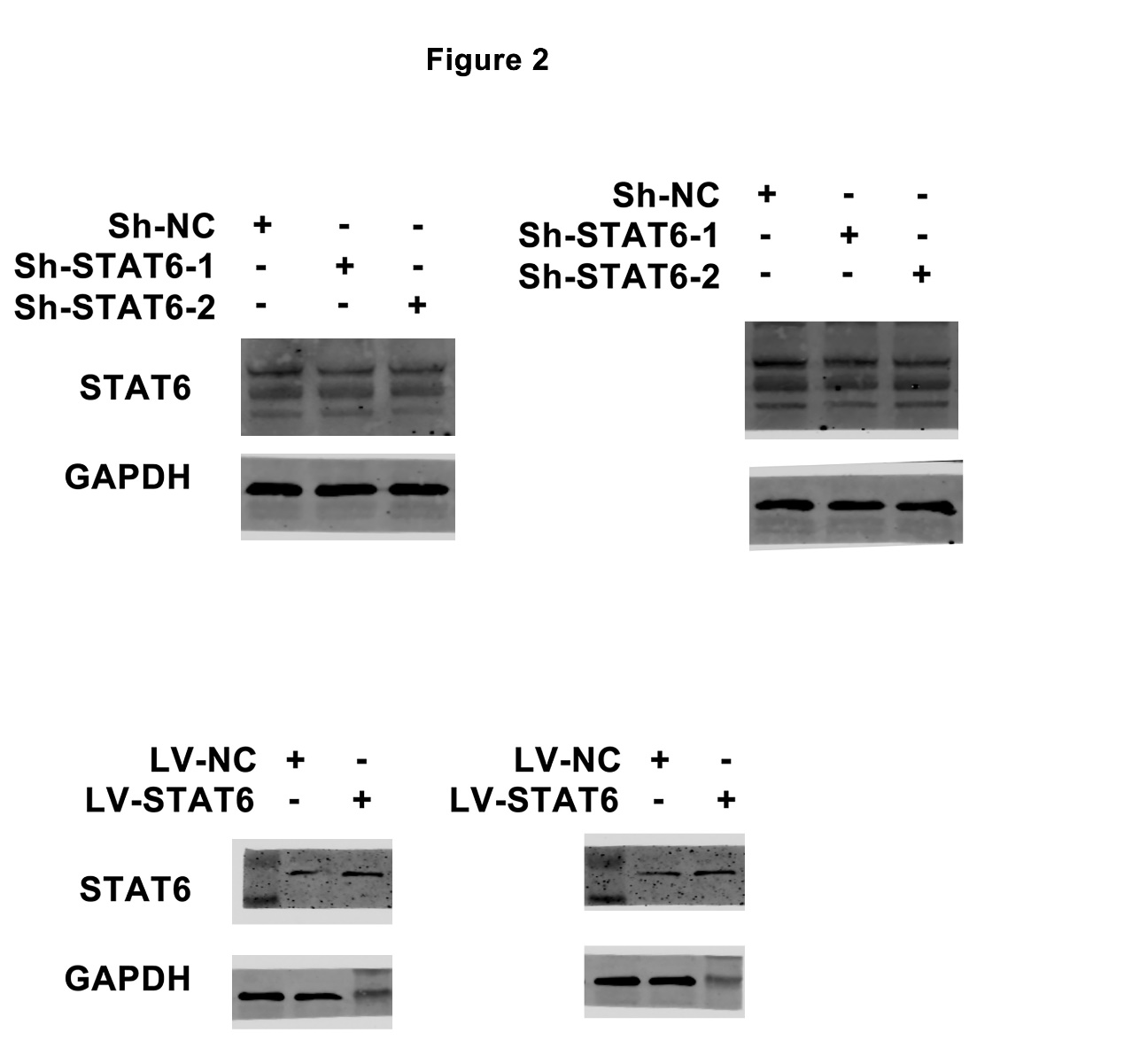


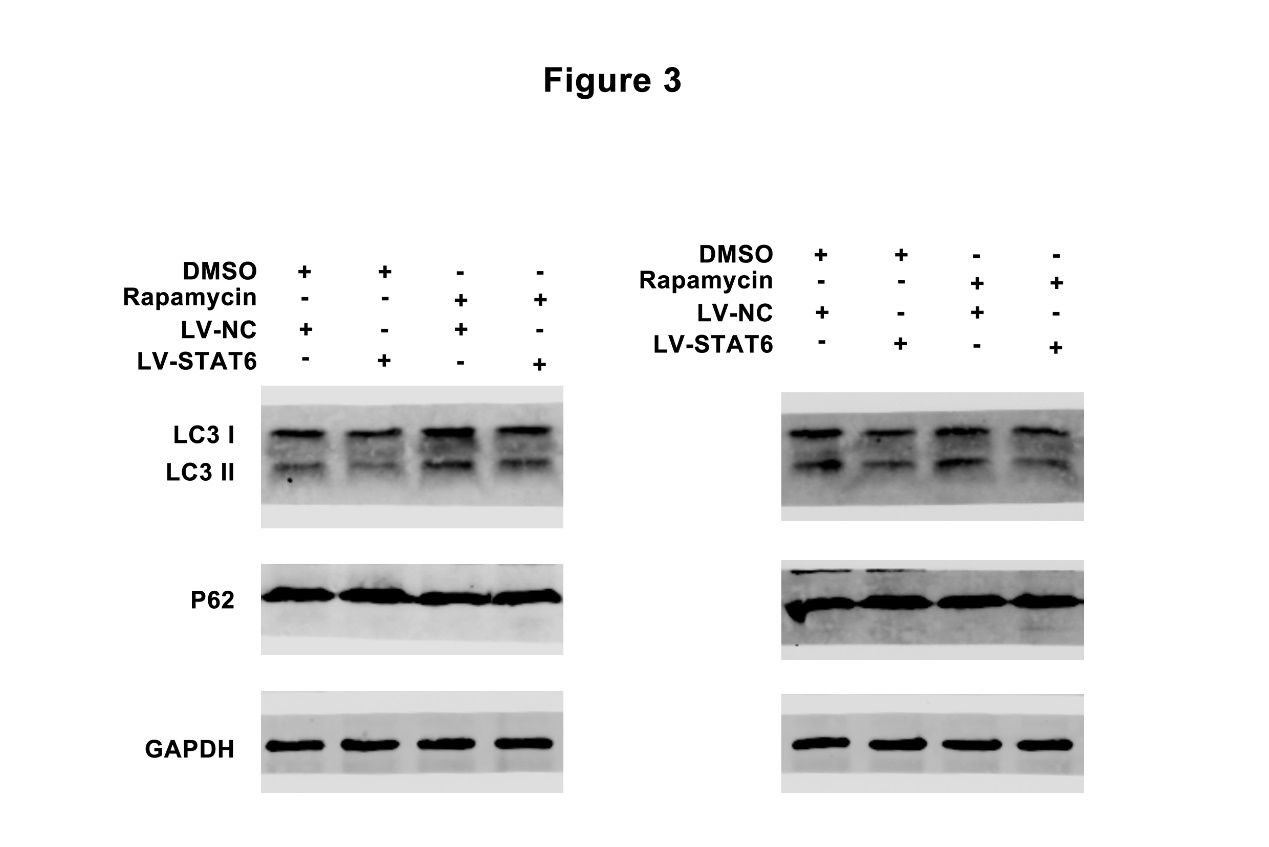


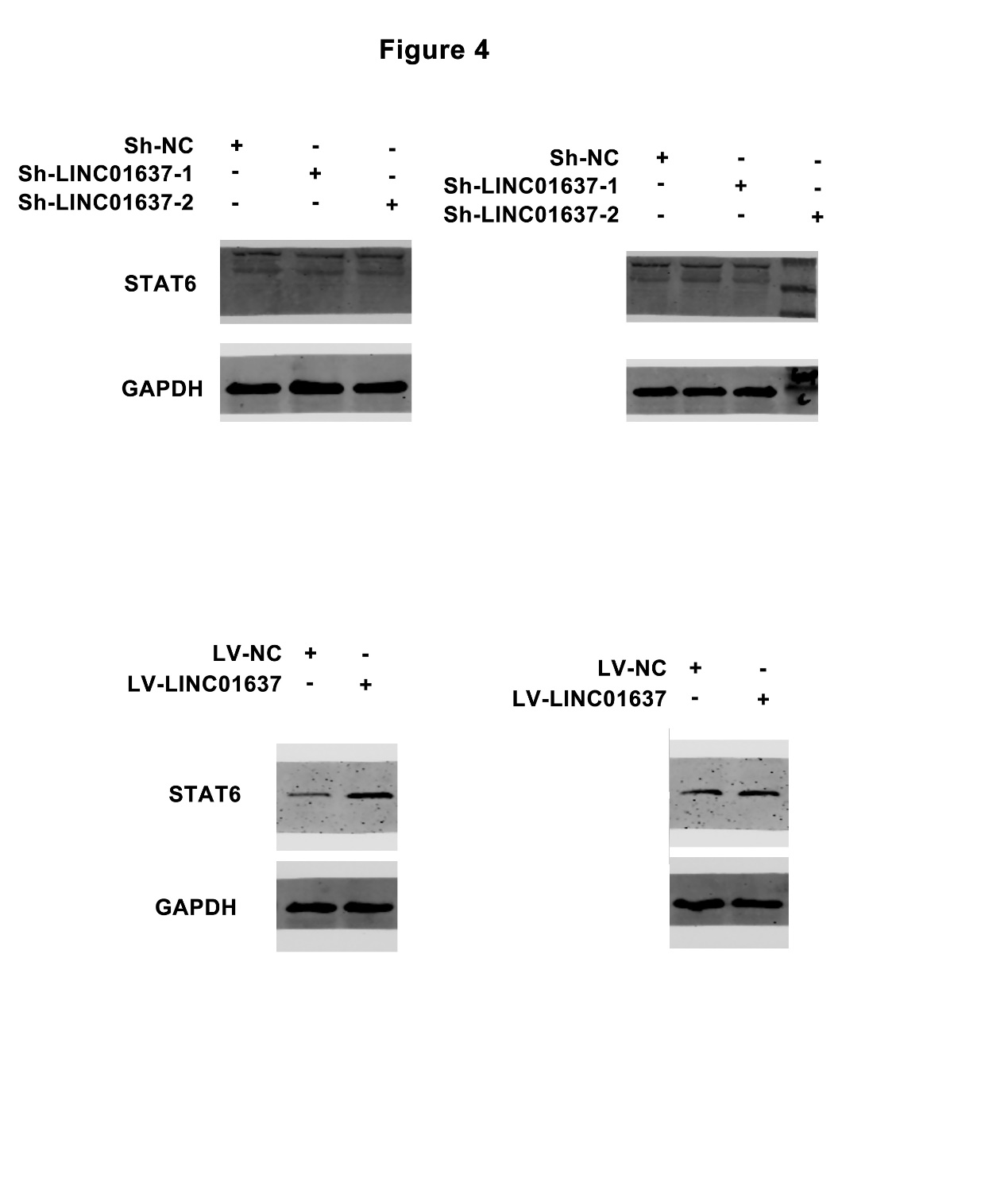


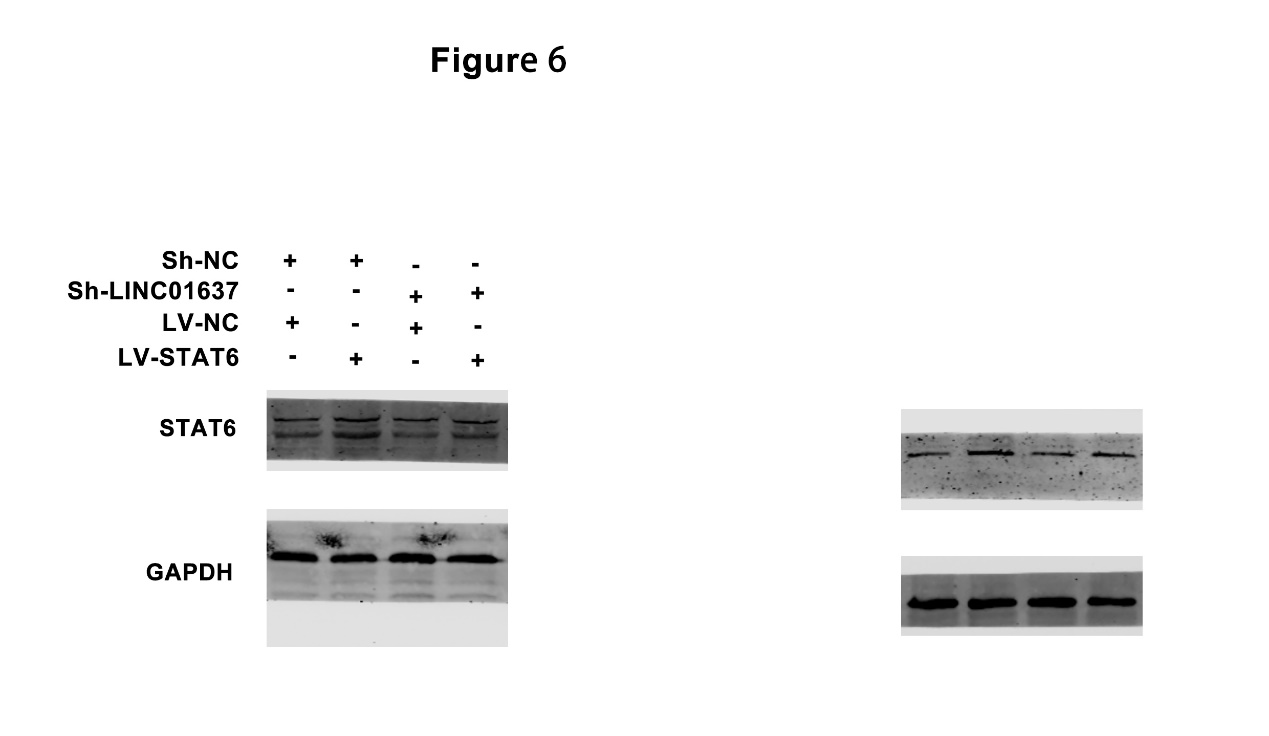


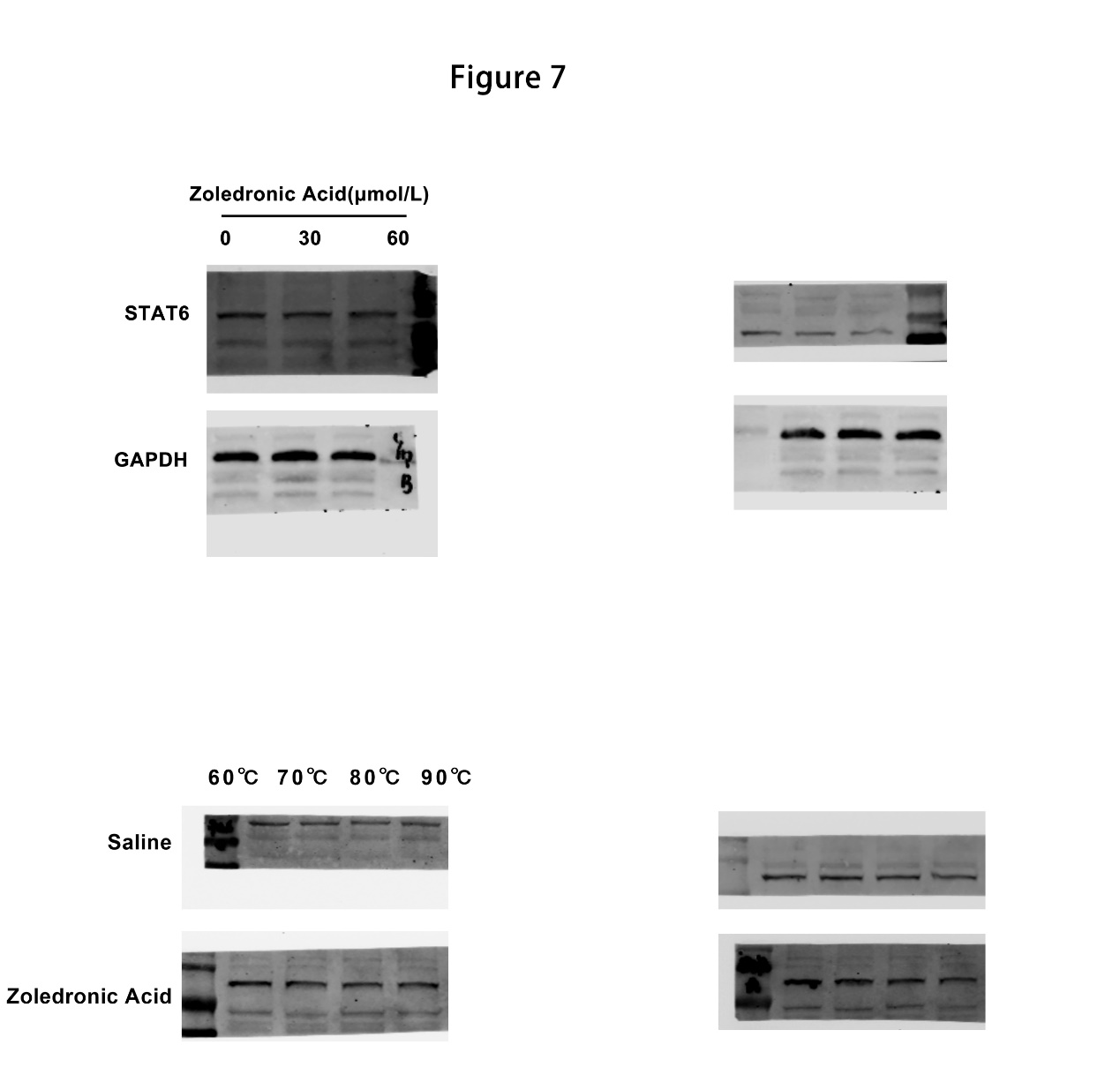


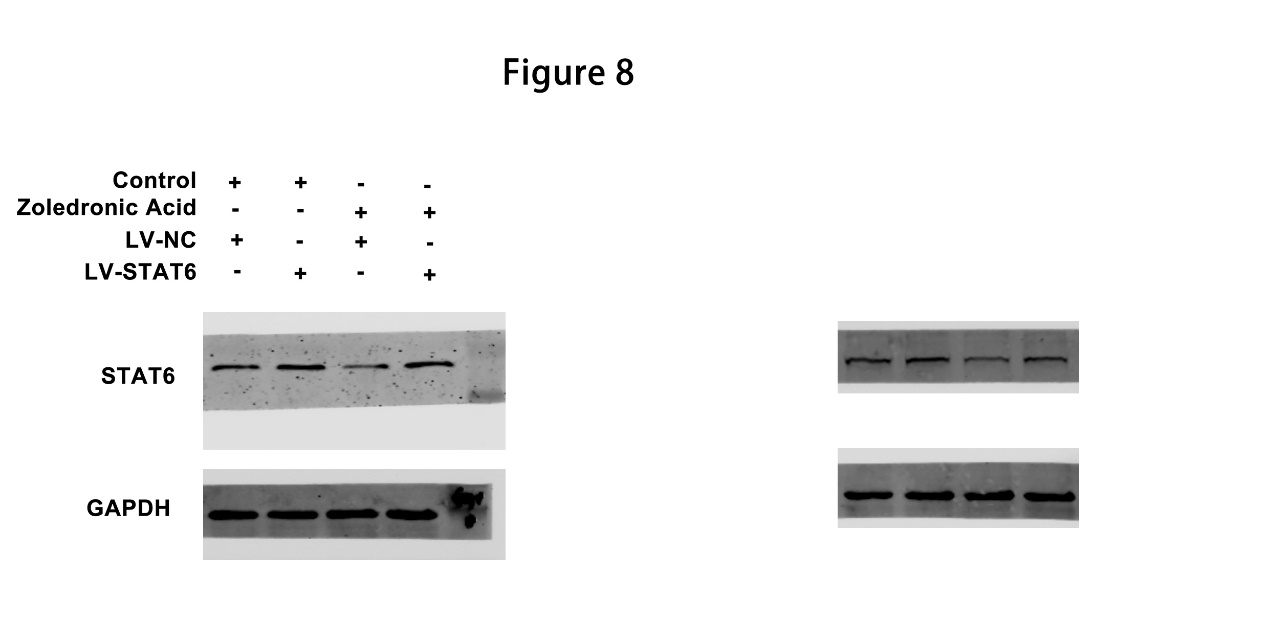


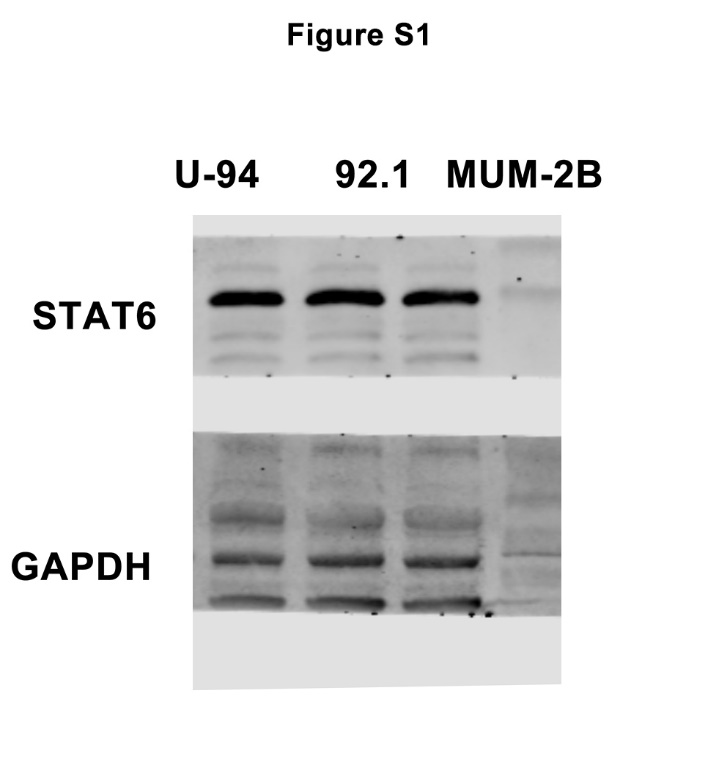


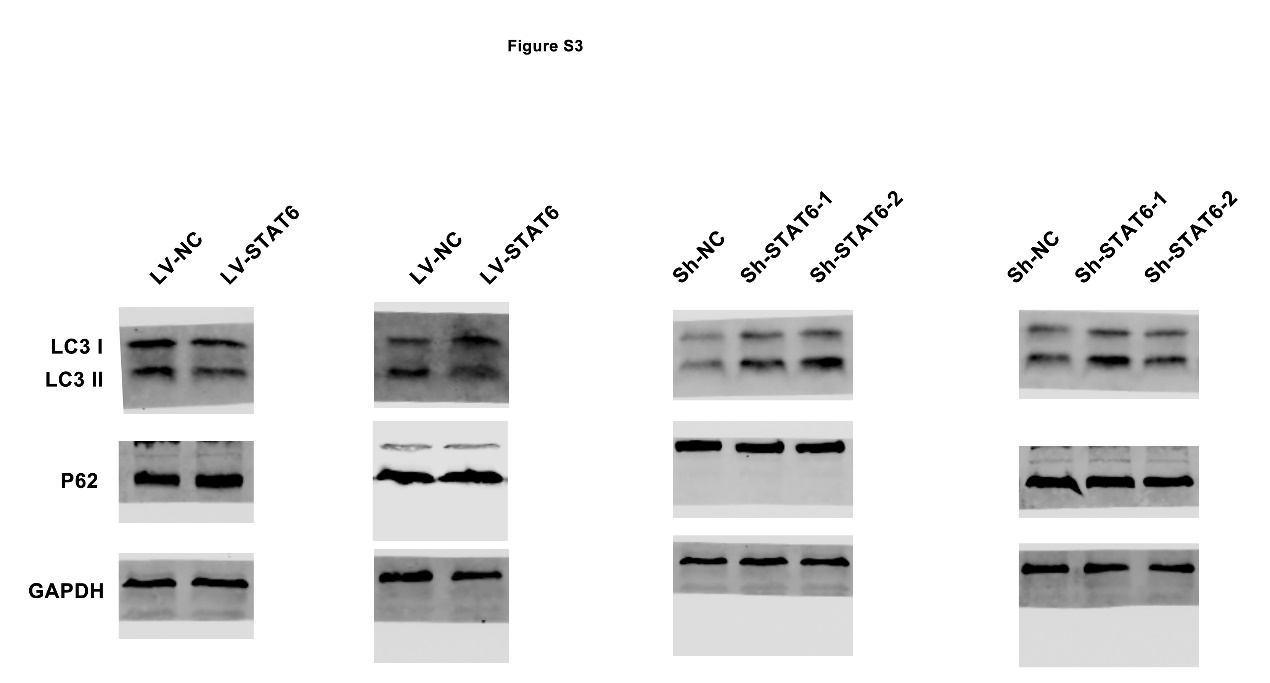


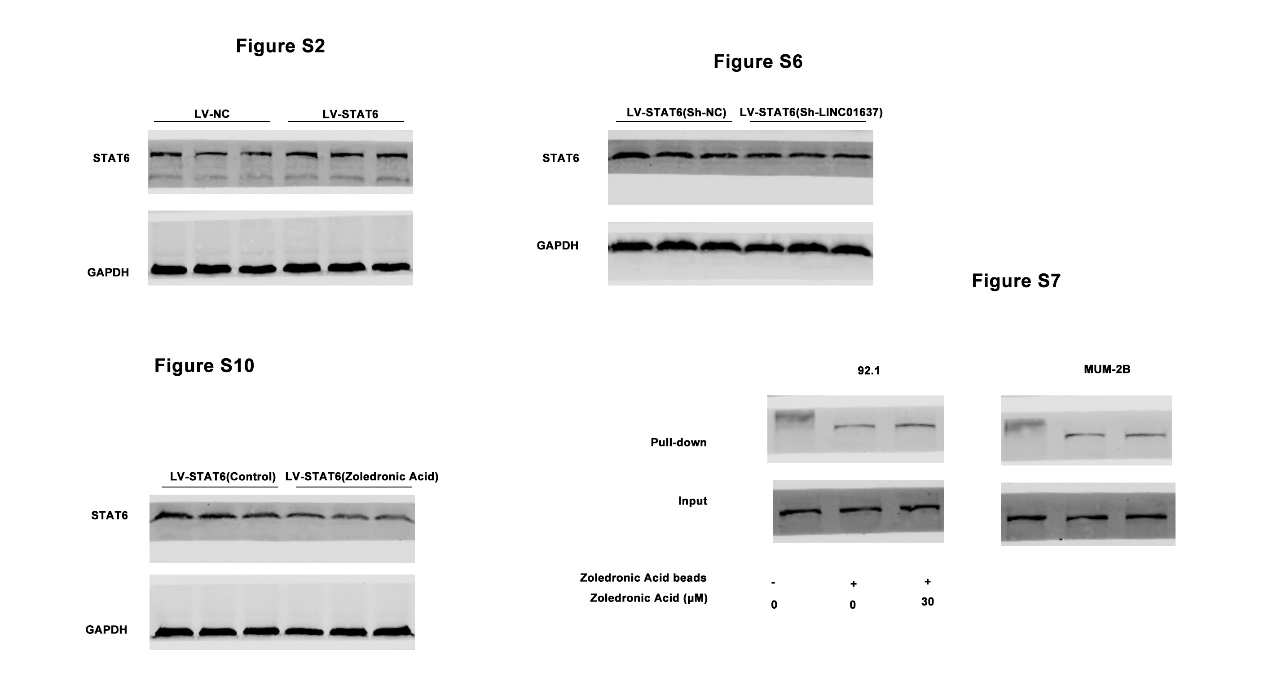

Supplement: Supplementary file 2 — Original Data [file 41419_2024_7115_MOESM2_ESM.docx]
